# Therapy-Induced Senescence (TIS) and SASP: The p53-Mediated Interplay in Cancer Progression and Treatment

**DOI:** 10.3390/ijms27010357

**Published:** 2025-12-29

**Authors:** Chang Hoon Lee, Tuan Minh Nguyen, Yongook Lee, Seoung Gyu Choi, Phuong Ngan Nguyen, Jung Ho Park, Mi Kyung Park

**Affiliations:** 1 BK21 FOUR Team and Integrated Research Institute for Drug Development, College of Pharmacy, Dongguk University, Goyang 10326, Republic of Korea; 2 Department of Biomedical Science, Hwasung Medi-Science University, Hwaseong-si 18274, Republic of Korea

**Keywords:** cellular senescence, p53, therapy-induced senescence (TIS), senescence-associated secretory phenotype (SASP), senolytics, senomorphics, cancer immunotherapy

## Abstract

Cellular senescence, initially regarded as a potent tumor-suppressive mechanism, is now recognized as a double-edged sword that modulates the hallmarks of cancer. The tumor suppressor p53 typically orchestrates this process to inhibit tumorigenesis; however, mutations in p53 or its regulators can subvert this program, leading to senescence evasion and therapy resistance. In particular, therapy-induced senescence can paradoxically drive tumor progression via the senescence-associated secretory phenotype, which creates a pro-tumorigenic microenvironment dictated by p53-mediated regulation of NF-κB signaling. Here, we explore the p53-mediated senescence–cancer interplay and evaluate emerging therapies, including senolytics and immunotherapies. We propose that strategic modulation of senescence offers a promising paradigm for future anticancer therapy.

## 1. Introduction

For decades, anticancer therapy has centered on inhibiting uncontrolled proliferation and inducing cellular demise of malignant cells. Conventional strategies—chiefly chemotherapy and radiotherapy—represent the cornerstones of this approach and have substantially extended the survival of countless patients.

Despite these advances, therapeutic resistance and subsequent tumor recurrence—driven by the persistence of residual cancer cells that survive treatment—remain the most formidable challenges in modern oncology. Overcoming this clinical impasse is paramount [1,2].

At the heart of this limitation lies cellular senescence. Defined as a state of stable and permanent cell cycle arrest, senescence is triggered in response to various forms of cellular stress or damage. It was initially recognized as a critical intrinsic defense mechanism that suppresses tumorigenesis, primarily induced by key tumor-suppressive pathways such as those governed by p53 (the guardian of the genome) and the p16/Rb (retinoblastoma protein) pathway [3].

However, emerging research has revealed that this protective barrier can paradoxically become a promoter of malignancy under specific conditions. A primary concern is therapy-induced senescence (TIS), which arises when cancer cells evade apoptosis following treatment and instead enter a stable senescent state.

These persistent senescent cells, often referred to as a distinct pathological identity, acquire a detrimental function by releasing a complex pro-inflammatory secretome known as the senescence-associated secretory phenotype (SASP). The SASP comprises a diverse array of inflammatory cytokines, growth factors, and proteases that actively remodel the local tumor microenvironment (TME). This remodeling creates a pro-tumorigenic niche that promotes tumor recurrence, angiogenesis, and malignant progression [4]. Therefore, elucidating the mechanisms of TIS and developing strategies to control it may hold the key to overcoming the profound limitations of existing anticancer therapies.

A central scientific controversy in modern oncology is whether therapy-induced senescence (TIS) represents a terminal success of anticancer treatment or a latent failure that seeds future recurrence. The major conceptual gap this review seeks to clarify is how the functional status of p53 dictates the transition of senescent cells from a tumor-suppressive state to a pro-tumorigenic driver. By establishing p53 as the master regulator of the SASP flavor, we provide a unifying framework to evaluate the clinical risks and therapeutic vulnerabilities of senescent cancer cells.

While cellular senescence has been the subject of recent discussion, this review uniquely distinguishes itself by focusing on the ‘p53-mediated bifurcation’ of cell fate—a critical switch that determines whether TIS leads to immune clearance or recurrence. Furthermore, we bridge a significant gap in the current literature by integrating the emerging field of cancer neuroscience, explicitly discussing the underappreciated interplay between SASP and the nervous system (e.g., perineural invasion) [5]. This multi-dimensional approach offers a novel, forward-looking framework for next-generation senescence-targeted therapies.

## 2. Understanding Cellular Senescence

Cellular senescence is not merely a passive, dysfunctional state resulting from cellular aging. On the contrary, it is a highly dynamic process actively orchestrated by the execution of specific genetic programs. Thus, a thorough comprehension of senescence itself is an essential prerequisite before discussing its intricate relationship with cancer.

### 2.1. Triggers and Master Regulatory Pathways of Cellular Senescence

Cellular senescence is triggered by a diverse array of stressors that ultimately converge on two major tumor-suppressive pathways.

The primary inducers include progressive telomere shortening associated with cell division (Replicative Senescence), activation of potent oncogenes such as RAS (rat sarcoma viral oncogene family; oncogene-induced senescence [OIS]), and significant cellular insults like DNA damage and oxidative stress, often resulting from radiation or chemical agents [6,7].

Regardless of the initial trigger, the senescent state is largely governed by two master regulator pathways. The first is the p53/p21 pathway. In response to stimuli such as DNA damage, p53 (the guardian of the genome) is activated, leading to upregulation of its downstream target, p21, which enforces cell cycle arrest [8,9]. The second is the p16^INK4a^/pRb pathway, which establishes a more robust and durable senescent state over time [10,11]. It is generally understood that the p53/p21 pathway is more critical for the initiation of senescence, whereas the p16^INK4a^/pRb pathway is essential for its long-term maintenance.

The central controversy within these regulatory axes lies in the rheostat model of p53 signaling. A major conceptual gap remains in understanding how the cell ‘chooses’ between transient, reversible arrest and permanent senescence. This review evaluates the hypothesis that the p53-p21 axis functions not as a binary switch, but as a dynamic sensor where the duration and intensity of p53 pulses—governed by complex feedback loops with MDM2—dictate the ultimate commitment to a senescent fate. Crucially, this implies that p53 functions as a molecular rheostat rather than a simple binary switch. Unlike the p16^INK4a^/Rb pathway, which enforces a static blockade, p53 dynamics—specifically the transition from oscillatory pulses to sustained activation—determine whether a cell undergoes reversible arrest or commits to the irreversible rewiring of senescence. This dynamic sensing capability allows p53 to integrate stress intensity with cell fate decisions.

#### 2.1.1. Key Regulators of the p53 Pathway: An Elaborate Balance

As the central switch for senescence induction, p53 activity is meticulously modulated by a diverse array of proteins. This intricate balance is a critical determinant of both tumorigenesis and the aging process [12].

The activity of p53 is suppressed by several negative regulators. The most important of these is MDM2 (mouse double minute 2 homolog). Through its E3 ubiquitin ligase activity, MDM2 promotes the ubiquitination of p53, targeting it for proteasomal degradation [13,14,15]. Notably, differences in MDM2 activity levels differentially regulate p53 fate. Low levels of MDM2 activity induce the monoubiquitination of p53, which acts as a critical signal for nuclear export of p53. Conversely, high levels of MDM2 activity promote the polyubiquitination of p53, leading to its degradation within the nucleus [16].

MDM4 (MDMX) is a homolog of MDM2. Although lacking intrinsic E3 ligase activity, MDM4 forms a heterodimer with MDM2 to enhance its function and directly inhibits the transcriptional activity of p53 [17,18].

Another regulator, SIRT1 (sirtuin 1), is an NAD^+^ (nicotinamide adenine dinucleotide)–dependent deacetylase that suppresses p53 activity by deacetylating specific lysine residues, thereby promoting cell survival [19,20]. SIRT1 regulates vital functions such as cellular metabolism, stress response, and aging by deacetylating various substrates, including p53. Although initially considered a tumor promoter because it inhibits the tumor suppressor p53, the role of SIRT1 remains debated owing to conflicting evidence suggesting it also functions as a tumor suppressor through other pathways [20].

The activation of p53 is carried out by various regulators. Typically, ARF (alternative reading frame tumor suppressor, p14^ARF^/p19^ARF^) expression is induced by oncogenic stress, such as RAS activation. ARF directly binds to MDM2, the key negative regulator of p53, inhibiting MDM2-mediated p53 degradation. This action effectively “liberates” and stabilizes p53 [21,22,23]. As such, p19^ARF^ is a tumor suppressor that inhibits cell proliferation through both p53-dependent mechanisms (by antagonizing the p53 inhibitor MDM2) and p53-independent mechanisms (by inducing genes such as the *Btg* (B-cell translocation gene) family) [24]. Another important regulator, p300/CBP (CREB-binding protein), consists of essential co-activators that respond to stress signals, such as DNA damage, by acetylating p53. Acetylation of p53 stabilizes the protein and maximizes its DNA-binding capacity, potently promoting the expression of senescence-inducing genes such as *p21* [25].

Thus, p53 activation is determined by a delicate balance between inhibitory (MDM2, MDM4, and SIRT1) and activating (ARF and p300/CBP) signals, and disruption of this balance is a key mechanism of carcinogenesis.

#### 2.1.2. Cellular Checkpoints Governing p53 Activation and Stabilization

Under basal physiological conditions, the tumor suppressor p53 is tightly regulated through a negative feedback loop involving the E3 ubiquitin ligase MDM2. MDM2 targets p53 for continuous proteasomal degradation, maintaining its levels at a low threshold [13]. Activation and stabilization of p53, therefore, depend principally on disruption of this p53–MDM2 interaction through distinct cellular checkpoints [14,23,26].

##### DNA Damage Response (DDR) Checkpoint

The DDR checkpoint represents the most canonical mechanism for p53 activation [8]. Upon detection of DNA double-strand breaks, ATM (ataxia telangiectasia mutated kinase) kinase is activated and phosphorylates Chk2 (checkpoint kinase 2) [27]. Concurrently, single-strand breaks or replication stress recruit ATR (ataxia telangiectasia and Rad3-related kinase) kinase, which activates Chk1. These kinase cascades phosphorylate p53 at specific N-terminal serine residues (e.g., Ser15, Ser20). These post-translational modifications induce a conformational change that sterically hinders MDM2 binding, leading to rapid p53 accumulation [14].

##### Oncogenic Stress Checkpoint

The oncogenic stress checkpoint serves as a fail-safe mechanism against hyperproliferative signaling. Aberrant activation of oncogenes such as RAS or MYC induces expression of the tumor suppressor p14^ARF^ (p19^Arf^ in mice) [28]. p14^ARF^ physically interacts with MDM2 and sequesters it within the nucleolus, thereby preventing MDM2 from degrading p53 in the nucleoplasm.

##### Nucleolar Stress Checkpoint

p53 acts as a critical sensor for the integrity of the protein synthesis machinery [21]. Disruption of ribosome biogenesis triggers the release of ribosomal proteins such as RPL5 and RPL11 from the nucleolus. These free ribosomal proteins bind to and directly inhibit the E3 ligase activity of MDM2, resulting in p53 stabilization [9].

##### Metabolic Stress Checkpoint

Cells monitor their energetic status through metabolic checkpoints. Under conditions of energy deprivation or nutrient stress, the AMPK (AMP-activated protein kinase) pathway is activated. AMPK promotes p53 stability by directly phosphorylating p53, thereby linking metabolic homeostasis to cell cycle control [9].

**Figure 1:**
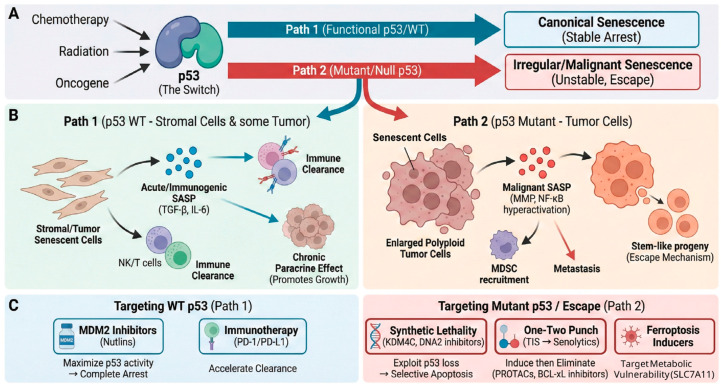
The dual pathways of cellular senescence determined by p53 status and corresponding therapeutic strategies. (**A**) p53 as a determinant switch in senescence. Upon stress signals such as chemotherapy, radiation, or oncogene activation, p53 directs the cell fate. Blue arrows indicate Path 1 (Functional/WT p53), leading to canonical senescence and stable cell cycle arrest. Red arrows indicate Path 2 (Mutant/Null p53), which initiates irregular or malignant senescence characterized by unstable genomic integrity and escape mechanisms. (**B**) Phenotypic and microenvironmental consequences of distinct senescence pathways. Path 1 (Blue) involves the secretion of acute/immunogenic senescence-associated secretory phenotype (SASP) factors like TGF-β and IL-6, promoting immune clearance by NK/T cells, although chronic persistence may promote tumor growth. Path 2 (Red) results in the formation of enlarged polyploid therapy-resistant senescent cells that secrete malignant SASP factors (driven by MMPs and NF-κB hyperactivation). This fosters an immunosuppressive environment (MDSC recruitment), metastasis, and the generation of stem-like progeny as an escape mechanism. (**C**) Therapeutic strategies targeting specific pathways. Interventions for Path 1 focus on maximizing WT p53 activity using MDM2 inhibitors (Nutlins) or accelerating immune clearance via immunotherapy (PD-1/PD-L1 blockades). Strategies for Path 2 (Mutant p53/Escape) exploit specific vulnerabilities through synthetic lethality (e.g., KDM4C or DNA2 inhibitors), a one-two punch approach (therapy-induced senescence followed by senolytics like PROTACs or BCL-xL inhibitors), or by targeting metabolic vulnerabilities (SLC7A11) with ferroptosis inducers.

The integration of these upstream signals determines the magnitude and duration of p53 activation [8]. Whereas transient stress from these checkpoints may trigger reversible cell cycle arrest for repair, sustained or overwhelming signaling drives p53 to induce irreversible fates such as apoptosis or cellular senescence, thereby suppressing tumorigenesis (Figure 1).

### 2.2. Characteristics of Senescent Cells: The Distinct Pathological Identity

Cells that have entered a state of cellular senescence exhibit complex characteristics beyond merely ceasing division. The most fundamental feature is irreversible growth arrest, wherein cells cannot re-enter the cell cycle even when external growth signals are present. Functionally, the most important characteristic is the SASP. Senescent cells actively secrete various substances, including cytokines, chemokines, growth factors, and proteases (matrix metalloproteinases), into the extracellular environment, exerting a powerful influence on the surrounding microenvironment. Although SASP can play beneficial roles in the acute phase, such as aiding wound healing or clearing early cancer cells [29,30], it also exhibits context-dependent characteristics. When chronically sustained, SASP instead plays a detrimental role by inducing inflammation and promoting cancer growth [31,32].

Furthermore, senescent cells undergo morphological and metabolic changes. They are characterized by increased cell size and a flattened appearance. Their lysosomal activity increases, causing them to exhibit a positive reaction to senescence-associated β-galactosidase staining [33,34]. Internally, permanent DNA damage sites condense within the nucleus to form distinctive structures called senescence-associated heterochromatin foci [35,36].

Importantly, these phenotypes are not limited to preclinical models but are clinically relevant. Histological analyses of tumor samples from patients undergoing neoadjuvant chemotherapy have frequently documented the accumulation of cells expressing high levels of p16^INK4a^ and senescence-associated β-galactosidase (SA-β-gal). Notably, the persistence of these senescent cells in residual disease has been significantly correlated with shorter recurrence-free survival and adverse clinical outcomes in breast and lung cancer cohorts, validating TIS as a critical pathological event in humans.

### 2.3. The Duality of Cellular Senescence

Cellular senescence acts as a double-edged sword, performing both beneficial and detrimental roles in the body. In the short term, it plays essential roles in suppressing tumorigenesis and aiding wound healing [37,38]. Upon liver damage, hepatic stellate cells enter senescence, which halts their proliferation and limits excessive liver fibrosis. Furthermore, these senescent cells secrete matrix-degrading enzymes and are preferentially eliminated by natural killer (NK) cells, thereby facilitating the resolution of fibrosis [37]. Senescent cells possess high immunogenicity, strongly activating dendritic cells and antigen-specific CD8^+^ T cells through alarmin release, interferon signaling activation, and enhanced MHC (major histocompatibility complex) class I machinery [38]. This immunogenicity is superior to standard immunogenic cell death, and senescent cancer cells can be used as a vaccine to induce potent and protective CD8-dependent antitumor immune responses.

**Figure 2:**
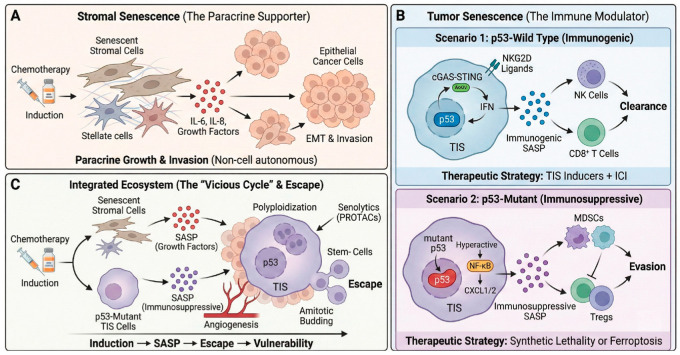
The distinct roles of stromal and tumor senescence in shaping the tumor microenvironment and therapeutic responses. (**A**) Stromal senescence as a paracrine supporter. Chemotherapy induction triggers senescence in stromal cells (e.g., stellate cells), leading to the secretion of SASP factors such as IL-6, IL-8, and growth factors. This paracrine signaling promotes epithelial–mesenchymal transition (EMT) and invasion in adjacent epithelial cancer cells via non-cell autonomous mechanisms. (**B**) Tumor senescence as an immune modulator determined by p53 status. Scenario 1 (p53-Wild Type): In cells with functional p53, therapy-induced senescence (TIS) activates the cGAS-STING-IFN axis. This upregulates NKG2D ligands and releases immunogenic SASP, recruiting NK cells and CD8+ T cells for tumor clearance. This context favors the combination of TIS inducers with immune checkpoint inhibitors (ICI). Scenario 2 (p53-Mutant): In cells with mutant p53, TIS leads to NF-κB hyperactivation and CXCL1/2 secretion. This results in an immunosuppressive SASP that recruits MDSCs and Tregs, facilitating immune evasion. Therapeutic strategies in this scenario require synthetic lethality or ferroptosis induction. (**C**) The integrated ecosystem and escape mechanism. Chemotherapy simultaneously induces senescence in the stroma and p53-mutant tumor cells, creating a vicious cycle. The combined secretome fosters angiogenesis and survival. Large polyploid TIS cells eventually undergo depolyploidization via amitotic budding, generating stem-like progeny that escape therapy. This vulnerability can be targeted by specific senolytics (e.g., PROTACs).

However, as senescent cells accumulate in the body with age, they promote systemic aging through chronic inflammation and become a cause of various chronic diseases, such as arteriosclerosis, diabetes, and neurodegenerative disorders [39,40]. A study utilizing the INK-ATTAC (INK-linked apoptosis through targeted activation of caspase) transgenic mouse model, which enables elimination of p16^Ink4a^-positive senescent cells, causally identified senescent cells as a key driver of age-related pathologies [40]. Furthermore, this study demonstrated that removal of these cells—even late in life—delayed the onset or attenuated the progression of established disorders, thereby extending healthspan. In the context of cancer therapy, it is precisely this detrimental aspect that contributes to treatment failure (Figure 2).

## 3. Interaction Between Cellular Senescence and the Hallmarks of Cancer

The influence of cellular senescence on cancer is most clearly understood through the conceptual framework of the “Hallmarks of Cancer.” Senescence plays a dual role, capable of both inhibiting and promoting these hallmarks depending on the context, with p53 functioning as the master regulator of this process. We examined the role that cellular senescence plays in the hallmarks of cancer.

### 3.1. Cellular Senescence: The Primary Barrier Against Carcinogenesis

Cellular senescence serves as a primary barrier against carcinogenesis by counteracting two key hallmarks of cancer.

First, senescence counteracts the evasion of growth suppression. When a potent oncogene such as RAS is activated, the cell activates p53 via the ARF–MDM2 pathway. Activated p53 subsequently induces its downstream target, p21, driving the cell into an irreversible cell cycle arrest known as OIS. This mechanism represents one of the most potent early defense mechanisms preventing cancer progression [28].

Second, senescence counteracts the acquisition of replicative immortality. Telomere attrition, which occurs during normal cell division, is recognized as DNA damage, thereby activating p53 and inducing replicative senescence [41]. This finding provided critical support for the hypothesis that telomere attrition functions as a replicative clock responsible for the finite doubling capacity of normal cells. Accordingly, replicative senescence acts as a critical barrier preventing the limitless proliferation of cancer cells.

### 3.2. TIS and SASP: Key Drivers of Malignancy

The SASP secreted by TIS cells that survive anticancer therapy paradoxically promotes the hallmarks of cancer. The functional status of p53 is a critical factor determining the nature of the SASP, often termed the SASP flavor.

**Figure 3:**
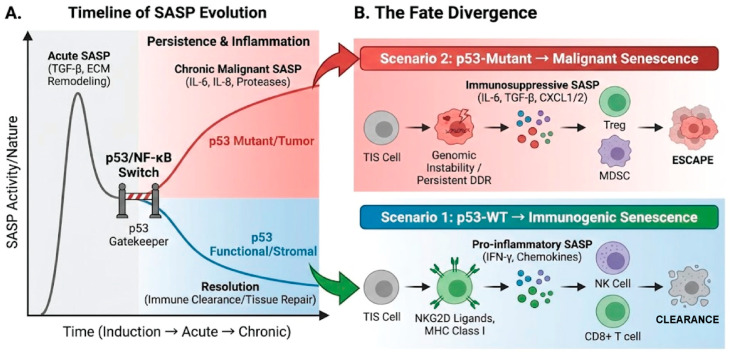
The p53-Mediated Bifurcation: Temporal SASP Evolution and Divergent Senescence Fates. (**A**) Timeline of SASP Evolution: The schematic illustrates the dynamic progression of the Senescence-Associated Secretory Phenotype (SASP) over time. Following an initial acute phase characterized by TGF-β secretion and extracellular matrix (ECM) remodeling, the p53/NF-κB axis functions as a molecular gatekeeper. Under functional p53 signaling (blue trajectory), the SASP transitions toward resolution, enabling immune clearance and tissue repair. In contrast, p53 mutation or a tumor-promoting environment (red trajectory) drives the SASP into a chronic, malignant state characterized by persistent inflammation and high levels of IL-6, IL-8, and proteases. (**B**) The Fate Divergence: This panel details the immunological outcomes of the p53-mediated bifurcation, where the color scheme distinguishes between tumor-suppressive (blue) and tumor-promoting (red) pathways: Scenario 1 (p53-WT → Immunogenic Senescence): Functional p53 induces an immunogenic phenotype in Therapy-Induced Senescent (TIS) cells. Upregulation of NKG2D ligands and MHC Class I molecules, combined with a pro-inflammatory SASP (IFN-γ, chemokines), recruits NK cells and CD8+ T cells for effective cell clearance. Scenario 2 (p53-Mutant → Malignant Senescence): Loss of functional p53 leads to genomic instability and a persistent DNA damage response (DDR). These cells secrete an immunosuppressive SASP (IL-6, TGF-β, CXCL1/2) that recruits Tregs and MDSCs, allowing senescent cells to escape immune surveillance and persist.

Importantly, SASP is not a static phenotype. Single-cell transcriptomic analyses reveal that the SASP evolves dynamically over time [42]. The early SASP is often TGF-β-rich and fibrotic, attempting to arrest cell growth, whereas the late SASP shifts towards a proinflammatory and matrix-degrading profile (interleukin-6 [IL-6], matrix metalloproteinases) driven by persistent NF-κB and cyclic GMP–AMP synthase (cGAS)–STING (stimulator of interferon gene) signaling. p53 plays a decisive role in this temporal switch. Functional p53 can restrain the transition to the late/malignant SASP by suppressing NF-κB activity [43]. However, p53 loss accelerates this shift, causing TIS cells to rapidly secrete a full-blown, protumorigenic secretome. Therefore, therapeutic strategies must consider intervention timing to target specific SASP phases. In this context, p53 acts as a critical temporal gatekeeper. Functional p53 actively restrains the shift from the Early SASP (TGF-β dominant, fibrotic) to the Late SASP (NF-κB dominant, pro-inflammatory). The loss of p53 removes this brake, accelerating the evolution toward a Malignant SASP that fuels tumor progression. Thus, p53 status dictates not only the growth arrest but also the qualitative flavor and temporal trajectory of the secretome (Figure 3A).

The rapid development of single-cell RNA sequencing (scRNA-seq) and spatial transcriptomics has fundamentally expanded our understanding of SASP heterogeneity. Although technical limitations persist, such multi-omic profiling has revealed that in vivo senescent cells are not a uniform population but exist as distinct clusters with divergent secretory profiles [34,44]. Indeed, studies from 2023 and 2024 have identified spatially distinct inflammatory versus fibrotic senescent subpopulations within the same tumor microenvironment, regulated by opposing NF-κB and TGF-β signaling networks. This molecular heterogeneity explains why conventional broad-spectrum senolytics often yield variable clinical responses, necessitating precision targeting of specific SASP subtypes [36]. In the future, the utilization of machine learning tools capable of integrating complex multi-omic datasets, alongside advanced algorithms like SenePy, will play a decisive role in overcoming current technical limitations and establishing personalized SASP modulation strategies tailored to individual patient [45,46].

It is crucial to recognize that the functional impact of TIS is highly context-dependent and varies by tumor histology. For instance, while senescent stromal fibroblasts typically generate a fibrotic and growth-promoting niche via TGF-β secretion, TIS in glioblastoma creates a distinct immunosuppressive cold microenvironment characterized by neuro-modulatory factors and exclusion of cytotoxic T cells. This lineage-specific heterogeneity dictates that therapeutic strategies must be tailored not only to the genetic driver (e.g., p53) but also to the tissue of origin.

Senescent cells induced by genotoxic stress, while arresting proliferation, acquire a SASP characterized by the secretion of factors associated with inflammation and malignancy. Significantly, this SASP can induce an epithelial–mesenchymal transition and invasiveness in nearby premalignant cells in a paracrine manner, often through factors such as IL-6 and IL-8. Moreover, these protumorigenic properties are markedly amplified and accelerated by functional loss of the tumor suppressor p53 or activation of oncogenic RAS. This suggests that p53 can suppress cancer through a cell-nonautonomous mechanism by modulating the surrounding tissue microenvironment [47].

Recently, the innate immune signaling pathway cGAS–STING has garnered attention as a key mechanism for SASP expression. This pathway is activated by cytoplasmic DNA (self-DNA [self-derived DNA]) that accumulates within senescent cells. Once activated, the pathway performs dual functions, either promoting or suppressing tumors depending on the biological context. Consequently, the cGAS–STING pathway is emerging as an important potential therapeutic target for modulating the cancer microenvironment [48].

However, this pathway presents a clinical paradox dictated by the duration of senescence. In the acute phase, cGAS–STING activation drives Type I interferon production, recruiting cytotoxic T cells for tumor clearance. In contrast, under chronic conditions, persistent cGAS signaling preferentially engages non-canonical NF-κB pathways, fueling a pro-tumorigenic SASP (e.g., IL-6, TNF-α) that fosters immune suppression and metastasis. Therapeutic targeting must therefore distinguish between these temporal ‘switches’ to avoid inadvertent tumor promotion [48,49]. A pivotal driver of this chronic inflammatory signaling is the reactivation of LINE1 retrotransposable elements. p53 plays a pivotal role in maintaining genomic stability by silencing LINE1 (L1) mobile elements, a process it achieves by cooperating with histone methyltransferases SETDB1 and G9A to deposit repressive H3K9me3 marks at the L1 promoter [50]. However, in p53-deficient senescent cells, the loss of repressive control leads to a ‘retrotransposition storm,’ resulting in the accumulation of cytosolic LINE1 cDNA [51]. This endogenous DNA species serves as a potent ligand for cGAS, sustaining a robust, interfron-driven SASP even in the absence of external DNA damage, linking genomic instability directly to chronic inflammation [48,49,52].

Critically, the SASP is not a static phenotype but evolves through distinct subtypes [42]. Single-cell profiling reveals that ‘early SASP’ is primarily fibrotic and growth-arresting, whereas late SASP shifts toward a malignant, proinflammatory program [42]. p53 serves as a decisive switch in this transition; functional p53 restrains the transition to a late/malignant SASP, while p53 loss or mutation accelerates it, promoting a pro-tumorigenic niche [43]. The cGAS–STING pathway illustrates a major conceptual paradox within this framework. While acute DNA damage sensing initially stimulates anti-tumor immunity through Type I interferons, persistent signaling in senescent cells chronically drives a pro-tumorigenic SASP (e.g., IL-6, MMPs) that facilitates immune evasion and metastasis [48,49]. Therapeutic strategies must therefore differentiate between these temporal immune signals to avoid inadvertently promoting tumor progression.

#### 3.2.1. Invasion and Metastasis

Senescence can also contribute to the activation of invasion and metastasis. For instance, hepatoma cells induced into senescence by therapy secrete large amounts of proteases, such as MMP-2 (matrix metalloproteinase-2), which degrade the surrounding extracellular matrix. Animal model studies confirmed that this matrix degradation significantly increases the invasive capacity of nearby cancer cells, thereby promoting metastasis [53]. The oncogene NFATc1 (nuclear factor of activated T cells c1), which is overexpressed in hepatocellular carcinoma, correlates with poor prognosis and drives tumor progression by enhancing cell proliferation, migration, and invasion [54]. Mechanistically, this protumorigenic function of NFATc1 is closely associated with amplification of the SASP—independent of growth arrest—and the NF-κB/TMP21 (transmembrane p24 trafficking protein 21) signaling pathway.

#### 3.2.2. Inducing Angiogenesis

When TIS was induced in breast cancer cells by treatment with the chemotherapeutic agent doxorubicin, these cells significantly increased their secretion of VEGF (vascular endothelial growth factor), promoting the proliferation of surrounding vascular endothelial cells and the formation of new blood vessels [55]. The poor prognosis of pancreatic cancer patients with comorbid diabetes is mechanistically linked to the expansion of senescent endothelial cells within the TME and their secretion of the TGF-β (transforming growth factor-β) family SASP factor INHBB (inhibin-β B subunit) [56]. INHBB promotes tumor progression, and therapeutic inhibition of its receptor with bimagrumab demonstrated effective antitumor responses in diabetic mouse models, suggesting INHBB as a novel therapeutic target for pancreatic cancer comanaged with diabetes.

#### 3.2.3. Sustaining Proliferative Signaling

SASP factors secreted by senescent fibroblasts promote the growth of nearby breast cancer epithelial cells in a paracrine manner, which can be a significant cause of cancer recurrence [57,58]. For example, senescent fibroblasts, through secreted factors such as the SASP (regardless of the inducing agent), stimulate the proliferation of premalignant and malignant epithelial cells in vitro and potently promote tumor formation by these cells in in vivo mouse models [57].

#### 3.2.4. Evading Immune Destruction

When the TIS state is chronically sustained, the SASP attracts immunosuppressive cells, such as myeloid-derived suppressor cells and regulatory T cells (Tregs), to the tumor. This results in suppression of cytotoxic T lymphocytes that are meant to attack cancer cells, fostering an immune-evasive environment [59].

GDF15 (growth differentiation factor 15) is emerging as a key biomarker for predicting systemic aging and all-cause mortality [60] and is also closely associated with chronic inflammation and immune decline in older adults [61,62]. Beyond these systemic roles, GDF15 functions as a critical secreted factor that suppresses antitumor immunity within the TME. The immunosuppressive function of GDF15 is mediated through two primary mechanisms. First, in cancers such as colorectal cancer and malignant glioma, GDF15 directly reduces the cytotoxicity of NK cells, helping cancer cells evade immune surveillance [63]. Second, GDF15 physically obstructs the infiltration of cytotoxic CD8^+^ T cells into tumor tissue [64]. This occurs because GDF15 inhibits the LFA-1 (lymphocyte function-associated antigen 1)/β2-integrin-mediated adhesion to vascular endothelial cells, which is essential for T cell trafficking—a mechanism confirmed in melanoma and other models.

### 3.3. SASP and Tumor Innervation: An Interaction

The recent emergence of cancer neuroscience highlights the intricate involvement of the nervous system in tumor growth and metastasis [65,66]. In this context, the SASP functions as a key molecular link mediating interactions among the nervous system, the TME, and cancer cells. SASP acts as a context-dependent, either inhibiting or promoting cancer depending on the context, and this dual nature is particularly evident in its relationship with the nervous system [65,67].

#### 3.3.1. Brain TME and Neuro-Immune Modulation

The detrimental impact of SASP on cancer progression is especially evident in brain tumors such as glioblastoma [65]. Glioblastoma is highly resistant to immune checkpoint inhibitors (ICIs), partly because of the potent immunosuppressive environment within the TME. Preclinical models suggest that SASP, particularly proinflammatory cytokines such as IL-6, is a key factor in reprogramming the microenvironment to be protumorigenic. These SASP factors contribute to a cold tumor environment by reinforcing an immunosuppressive state, thereby rendering ICIs ineffective. Interestingly, a novel therapeutic approach has been proposed to suppress these detrimental effects of SASP by leveraging the nervous system itself. Vagus nerve stimulation (VNS) can activate the body’s inflammatory reflex, which in turn can suppress the systemic and local secretion of key SASP components, including IL-6, IL-1β, and tumor necrosis factor-α. According to one hypothesis, combining VNS with ICIs could suppress SASP, converting an immune-resistant cold tumor into an immune-responsive hot tumor, thereby halting glioma progression. This presents a new therapeutic perspective, viewing cancer as a form of immune dysautonomia that can be reset via VNS [65].

#### 3.3.2. Direct Effects of SASP on Nerve Cells and Cancer Cells

Beyond modulating the brain TME, SASP exerts direct and significant effects on nerve cell and cancer cell phenotypes. First, SASP can induce neuronal toxicity. Astrocytes, the most abundant cell type in the brain, can be driven into senescence by treatments such as X-irradiation. These senescent astrocytes are characterized by specific downregulation of genes encoding glutamate and potassium transporters [68]. This dysregulation leads to collapse of glutamate homeostasis, ultimately causing excitotoxicity in cocultured neurons and leading to their death—a proposed mechanism for neurodegenerative diseases. Second, SASP inhibits nerve growth. For example, the chemotherapy agent cisplatin can induce senescence (identified by increased p16, p21, and p53) in cavernous nerves of rat models [69]. In vitro experiments also confirmed that the supernatant (SASP) secreted from senescent Schwann cells (RSC96) significantly slowed neurite outgrowth of healthy nerve ganglia [69]. Finally, SASP can directly alter cancer cell phenotypes by inducing neuroendocrine differentiation (NED). NF-κB–dependent SASP factors secreted from senescent cells trigger neuroendocrine transdifferentiation (NED) in breast and prostate epithelial cancer cells, causing them to acquire neuron-like characteristics [70]. This mechanism is mediated by an increase in intracellular calcium (Ca^2+^) signaling induced by the SASP. Critically, this neuro-phenotypic switch (NED) is not a mere side effect of TIS but represents a strategic rewiring of the TME. A significant knowledge gap exists regarding how p53-mutant malignant SASP specifically facilitates this transdifferentiation to bypass standard hormonal and cytotoxic therapies. By linking cancer neuroscience to the p53-SASP framework, we identify a critical controversy: whether targeting tumor innervation can effectively silence the pro-tumorigenic signals of senescent cells.

#### 3.3.3. SASP, Nerve-Related Cells, and Cancer Metastasis

The interaction between the nervous system and cancer culminates in perineural invasion (PNI), wherein cancer cells invade along nerves [5,71,72]. Tumor-associated Schwann cells, the most abundant glial cells in the peripheral nervous system, are key drivers of PNI and promote tumor recurrence and poor prognosis. Given that senescent Schwann cells (RSC96) secreted SASP in response to cisplatin, SASP secreted by senescent tumor-associated Schwann cells plausibly represents a key mechanism promoting PNI [69].

Conversely, proteins related to the nervous system can also suppress cancer. The neurotransmitter-releasing protein DOC2B (double C2-like domain-containing protein B) functions as a metastatic suppressor by inhibiting the epithelial–mesenchymal transition and inducing senescence [73].

**Figure 4:**
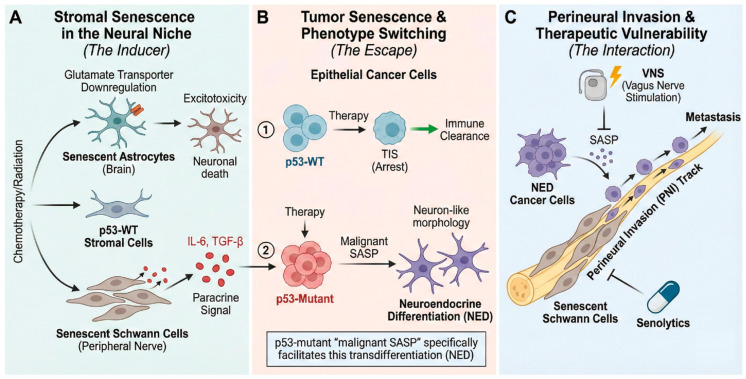
Stromal senescence in the neural niche drives tumor phenotype switching and perineural invasion (PNI). (**A**) Senescence induction in the neural niche. Therapy (chemotherapy/radiation) triggers senescence in astrocytes and Schwann cells. Senescent astrocytes downregulate glutamate transporters, leading to excitotoxicity and neuronal death. Concurrently, senescent Schwann cells secrete paracrine SASP factors, including IL-6 and TGF-β. (**B**) Tumor senescence and phenotype switching as an escape mechanism. While p53-wild type (WT) cells undergo therapy-induced senescence (TIS) and subsequent immune clearance, p53-mutant cells exploit malignant SASP to undergo Neuroendocrine Differentiation (NED). This transdifferentiation confers a neuron-like morphology, allowing the cells to escape therapy. (**C**) Perineural invasion dynamics and therapeutic vulnerabilities. Senescent Schwann cells create a permissive microenvironment via SASP, facilitating the invasion and metastasis of NED cancer cells along the perineural track. This axis presents therapeutic targets: Vagus Nerve Stimulation (VNS) to inhibit SASP secretion, or senolytics to eliminate the senescence-driving Schwann cells.

From the perspective of cancer neuroscience, cellular senescence and SASP are key elements that modulate the cancer microenvironment. They can induce neurotoxicity (via astrocytes), inhibit nerve growth (via Schwann cells), alter cancer cell phenotypes (NED), mediate metastatic processes such as PNI, and simultaneously serve as targets for novel neuromodulatory therapies like VNS (Figure 4).

### 3.4. p53 Mutation and Cellular Senescence: Evasion, Incompleteness, and Malignancy

Functional loss of p53, the most commonly mutated tumor suppressor gene, fundamentally alters the process of cellular senescence itself. This functional dichotomy leads to two divergent senescence fates: immunogenic senescence driven by wild-type p53, which recruits immune clearance, versus malignant senescence driven by mutant p53, characterized by genomic instability, immune evasion, and escape mechanisms. This fundamental rewiring explains why TIS can be curative in some contexts but promotes recurrence in others (Figure 3B).

#### 3.4.1. Senescence Evasion (Bypass)

In the early stages of carcinogenesis, p53 mutation plays a decisive role, allowing cells to completely bypass key defense mechanisms such as OIS or replicative senescence and to acquire immortal proliferative capacity [28,74]. For example, tumor dormancy in breast cancer models involves p53 activation, and tumors that escape dormancy possess p53 mutations [75]. This exit from dormancy requires both p53 inactivation and a permissive TME. Similarly, gain-of-function (GOF) p53 mutations, such as p53(N236S), can drive bypassing of HRasV12 (Harvey rat sarcoma viral oncogene V12)–induced senescence by increasing PGC-1α (peroxisome proliferator-activated receptor gamma coactivator-1α), enhancing mitochondrial biosynthesis, and inhibiting reactive oxygen species [76].

#### 3.4.2. Incomplete Senescence and Escape

Even if cells lacking functional p53 are forced into senescence by strong stress, such as chemotherapy, via p53-independent pathways (e.g., p16/pRb), this senescent state is often incomplete and unstable [77]. This instability can lead to senescence escape, a phenomenon wherein a subset of cells re-enters the cell cycle over time, acting as a direct cause of cancer recurrence [78].

Although this review primarily focuses on the p53 axis, the p16^INK4a^/Rb pathway remains a parallel and essential gatekeeper of senescence maintenance. In many clinical cases, the concomitant loss of p16 alongside p53 mutation accelerates the senescence escape phenotype, leading to a more aggressive and therapy-resistant recurrence than p53 loss alone.

Studies in p53 wild-type (MCF-7 ell line]) cells show that even these cells can escape TIS by undergoing polyploidization and subsequent atypical, amitotic divisions. This escape is even more pronounced in p53-deficient cells. For instance, certain mutant p53 isoforms (e.g., Δ133p53αR273H) actively reduce cellular senescence in an inducer-dependent manner (e.g., rescuing radiation-induced TIS but not temozolomide-induced TIS), thereby promoting aggressiveness [79]. TIS escape is not a monolithic phenomenon but displays significant phenotypic heterogeneity [79]. Some cells convert into polyploid giant cancer cells (PGCCs), capable of generating stem-like progeny via amitotic budding [79,80]. Others exhibit transient senescence, repairing damage to re-enter the cell cycle, or exist as cycling senescence-like cells that express senescence markers yet retain slow proliferative capacity [80]. This heterogeneity is a primary driver of therapeutic failure, enabling specific subpopulations to evade marker-dependent senolysis [12]. Consequently, when these cells enter a TIS state, they secrete a much more potent and protumorigenic malignant SASP [47,80].

Recent paradigm shifts have fundamentally updated our understanding of cellular aging. The authoritative 2023 review on the hallmarks of aging redefines senescence not as an irreversible endpoint, but as a dynamic state linked to disabled macroautophagy and chronic inflammation, which can be modulated [51].

Emerging evidence from 2022 to 2024 highlights senescence plasticity in oncology, where therapy-induced stress drives tumor cells into reversible dormant states, including diapause-like persister phenotypes or polyploid giant cancer cells (PGCCs) [81,82]. These persister/PGCC populations exhibit stem-like properties, evade senescence through mechanisms such as polyploidization, asymmetric division, amitotic budding, or depolyploidization, and generate aggressive, resistant progeny upon stress resolution—representing a key driver of treatment resistance and recurrence [82,83,84].

On the rejuvenation front, attention has shifted from mere senolysis to harnessing cellular plasticity for reversal. A landmark 2023 study demonstrated that chemical cocktails (e.g., valproic acid, CHIR99021) can reverse epigenetic aging markers and restore nucleocytoplasmic compartmentalization without genetic alteration [85]. This aligns with findings from Altos Labs (2022), which showed that partial reprogramming in vivo can ameliorate age-associated molecular changes [86]. Collectively, these advances (2022–2024) signal a transition toward precisely modulating reversible senescence and plasticity, supporting the geroscience hypothesis while offering new avenues for preventing age-related diseases and overcoming therapy resistance in cancer.

This phenomenon of senescence escape challenges the traditional definition of senescence as permanent arrest. The scientific controversy revolves around whether these escaped cells represent a unique subpopulation or result from an incomplete p53-mediated program. Furthermore, a significant knowledge gap exists regarding how p53 gain-of-function (GOF) mutations, such as R175H, actively reprogram the SASP into a potent immunosuppressive program that facilitates this escape.

#### 3.4.3. Malignant SASP and GOFs

A more severe problem arises when cancer cells harboring mutated p53 enter a TIS state. Wild-type p53 normally performs a regulatory function by suppressing certain SASP components (e.g., via p53-dependent regulation of ePGE1); however, p53-mutant cells lack this control. Consequently, these cells secrete a far more potent and protumorigenic malignant SASP. Crucially, the impact on SASP varies significantly by the mutation’s structural class. Conformational mutants (e.g., R175H) induce global protein unfolding, allowing mutant p53 to sequester other tumor suppressors like p63/p73 and co-activate NF-κB, thereby driving the most pro-inflammatory SASP enriched in IL-6, IL-8, and proteases [87]. Conversely, DNA-contact mutants (e.g., R273H) retain structural integrity but lose specific binding affinity, orchestrating a distinct transcriptomic program and metabolic rewiring that differs from conformational counterparts [79]. This phenomenon is exemplified in zebrafish models, where a GOF p53(R175H) mutation converts Ras-induced senescence from a tumor-suppressive mechanism into a tumor-promoting one [87]. Double-mutant cells survive, senesce, and secrete SASP factors that convert neighboring normal cells into senescent, SASP-secreting cells, generating a heterogeneous tumor-like mass. These findings align with clinical observations that *TP53* (tumor protein p53 gene) mutations are associated with higher histopathological grades in astrocytoma and are prevalent in early gastric lesions, alongside elevated SASP (cGAS–STING) and DDR markers [49].

Recent studies (2022–2025) have established that TP53 mutations, particularly gain-of-function (GOF) mutants, orchestrate an immune-privileged sanctuary in tumors by reshaping the tumor microenvironment (TME) toward immunosuppression.

Mutant p53 actively drives a distinct immunosuppressive senescence-associated secretory phenotype (SASP) program, hijacking pathways such as NF-κB to upregulate chemokines like CCL2, CXCL1, and CXCL5. This promotes recruitment of immunosuppressive tumor-associated macrophages (TAMs), neutrophils, and myeloid-derived suppressor cells while excluding cytotoxic CD8^+^ T cells—often through downregulation of T-cell attractants—resulting in an immune desert phenotype that protects residual disease and confers resistance to immune checkpoint inhibitors [88].

However, breakthrough 2025 findings reveal allele-specific vulnerabilities. Contact mutants (e.g., certain hot-spot variants) paradoxically induce massive replication stress by overriding the TopBP1/Treslin regulatory switch, leading to over-firing of replication origins in late S/G2 phases, micronuclei accumulation, and forced cGAS-STING activation. This renders these tumors sensitive to immune checkpoint blockade [89].

Additionally, low-dose statins pharmacologically degrade mutant p53, relieving its blockade on TBK1, restoring IRF3 phosphorylation/nuclear translocation, and promoting CD8+ T-cell infiltration [90].

Collectively, these insights support precision medicine approaches: exploiting replication stress vulnerabilities (e.g., via TopBP1-targeted or PARP inhibitors) in contact mutants, or restoring innate immunity (via statins or STING agonists) to dismantle the mutant p53-driven immunosuppressive sanctuary and overcome therapy resistance.

### 3.5. Metabolic Rewiring and Ferroptosis Resistance

Although senescent cells cease division, they exhibit a hypermetabolic state to support the extensive protein synthesis required for SASP production. Recent studies demonstrate that senescent cells accumulate high levels of intracellular iron and reactive oxygen species, rendering them intrinsically vulnerable to ferroptosis, an iron-dependent form of cell death [91,92,93].

Interestingly, p53 functions as a rheostat in this process. Wild-type p53 typically promotes ferroptosis by inhibiting SLC7A11 (solute carrier family 7 member 11, a component of the cystine/glutamate antiporter system xC−), thereby sensitizing cells to oxidative stress [94]. However, in p53-mutant tumors, *SLC7A11* is often upregulated, conferring resistance to ferroptosis and enabling these cells with distinct pathological identity to persist. Targeting this metabolic vulnerability—specifically by inducing ferroptosis in senescent cells (senolysis via ferroptosis)—is emerging as a novel therapeutic strategy that bypasses apoptosis resistance.

Multiple pharmacological approaches target senescence-associated pathologies. p53 reactivators such as Nutlin-3 (MDM2 inhibitor) and APR-246 (mutant p53 reactivator) restore tumor suppressor function to eliminate senescent cells or prevent malignant transformation. Senolytics, including Navitoclax (BCL-2 family inhibitor) and quercetin (natural flavonoid), selectively induce apoptosis in senescent cells. Senomorphics such as rapamycin (mTOR inhibitor) and ruxolitinib (JAK1/2 inhibitor) suppress the proinflammatory and protumorigenic SASP without killing cells. Synthetic lethality approaches such as QC6352 (KDM4C inhibitor) exploit specific vulnerabilities in senescent cells for selective cytotoxicity. Immunotherapies represent emerging strategies: PD-1/PD-L1 checkpoint inhibitors enhance T cell recognition of senescent cells expressing immunogenic markers, while CAR-T cells engineered with uPAR-specific recognition can selectively target and eliminate senescent populations.

**Figure 5:**
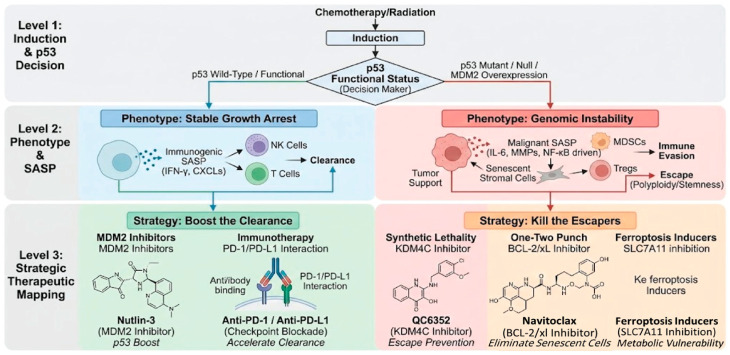
A hierarchical therapeutic decision tree based on p53 functional status. (Level 1: Induction & Decision) Upon stress induction by chemotherapy or radiation, the functional status of p53 (Wild-Type vs. Mutant/Null/MDM2 Overexpression) serves as the critical binary switch determining cell fate. (Level 2: Phenotype & SASP) Functional p53 drives a phenotype of stable growth arrest accompanied by an immunogenic SASP (IFN-g, CXCLs), facilitating immune clearance by NK and T cells. Conversely, dysfunctional p53 leads to genomic instability and a malignant SASP (IL-6, MMPs, NF-kB-driven). This creates an immunosuppressive microenvironment by recruiting MDSCs and Tregs, promoting tumor escape mechanisms such as polyploidy and stemness. (Level 3: Strategic Therapeutic Mapping) Therapeutic interventions are tailored to the specific senescence pathway. For p53-WT tumors, the strategy is to boost the clearance using MDM2 inhibitors (e.g., Nutlin-3) to enhance p53 activity or immunotherapy (PD-1/PD-L1 blockade) to accelerate elimination. For p53-Mutant tumors, the strategy is to kill the escapers utilizing synthetic lethality (e.g., KDM4C inhibitors/QC6352), a one-two punch senolytic approach (e.g., BCL-2/xL inhibitors/Navitoclax), or ferroptosis inducers targeting metabolic vulnerabilities (SLC7A11 inhibition).

Ultimately, this metabolic and signaling rewiring creates distinct therapeutic windows. The hypermetabolic and lipid-peroxidation-prone state of p53-mutant malignants senescence renders these cells uniquely susceptible to ferroptosis inducers and synthetic lethality strategies, forming the biological basis for the stratified decision tree outlined in Figure 5.

## 4. Novel Anticancer Therapeutic Strategies Targeting Cellular Senescence

As the specific malignant contributions of TIS cells have been elucidated, novel anticancer strategies aimed at precisely controlling these cells are being actively developed. Critically, these strategies cannot be applied indiscriminately; rather, their clinical utility depends on a precision medicine approach that stratifies patients based on the distinct molecular vulnerabilities created by the tumor’s p53 functional status (Figure 5).

### 4.1. Strategy 1: Reactivating the p53 Barrier—p53 Activators

This strategy does not involve inducing or utilizing TIS; rather, it represents a fundamental approach to induce senescence or apoptosis by repairing the p53 pathway within the cancer cell itself.

#### 4.1.1. MDM2/MDM4 Inhibitors

Many cancers suppress p53 by overexpressing MDM2 or MDM4, rather than harboring p53 mutations [95]. Small-molecule compounds of the Nutlin series bind to the p53-binding pocket of MDM2, thereby liberating p53 [96]. This reactivated p53 potently induces senescence or apoptosis in cancer cells through p21 expression [97]. Recently, dual inhibitors targeting both MDM2 and MDM4 (e.g., ALRN-6924) have shown promising results in clinical trials [98]. A first-in-human phase 1 trial involving patients with solid tumors and lymphomas demonstrated that, while the primary endpoint was safety, the drug also exhibited notable preliminary efficacy. The maximum tolerated dose was established at 3.1 mg/kg on the once-weekly schedule. Crucially, the treatment achieved a disease control rate of 59% specifically in TP53-wild-type patients. This result is significant as it provides clinical proof-of-concept that dual inhibition can effectively reactivate p53 to induce tumor growth arrest, although further phase 2 studies are required to confirm therapeutic benefit [98]. Despite this promising efficacy, hematological toxicities such as thrombocytopenia remain a significant clinical challenge, necessitating careful dose-titration strategies and patient monitoring to maintain an optimal therapeutic window.

#### 4.1.2. Mutant p53 Reactivators

Certain drugs restore mutant p53 protein function by reverting its tertiary structure to one resembling that of wild-type p53. APR-246 (eprenetapopt) is a representative agent with this mechanism; it induces radiosensitivity in *TP53*-mutant cancer cells and has been proposed as a promising strategy because it increases tumor cell death, particularly when combined with alpha-particle radiation therapy [99].

### 4.2. Strategy 2: Targeted Elimination—Senolytics

One promising therapeutic approach involves targeting senescent cells, also termed distinct pathological identity cells. p53 activation–induced senescence leads to cell-cycle arrest but simultaneously increases expression of BCL-2 (B-cell lymphoma 2) family antiapoptotic proteins, which promote cell survival rather than apoptosis [100]. This mechanism creates a survival dependency that can be therapeutically exploited. Senolytics are drugs designed to selectively eliminate senescent cells by targeting precisely this acquired vulnerability [40].

A key application of this concept is the one-two punch strategy. This sequential approach first involves inducing widespread senescence in cancer cells using a primary therapeutic agent (Step 1), followed by administration of a senolytic drug to selectively eliminate the induced senescent cell population (Step 2). The efficacy of this strategy has been demonstrated across various TP53 statuses. For example, in TP53-wild-type ovarian clear cell carcinoma, senescence could be induced with an MDM4 inhibitor (CEP-1347), after which senescent cells could be eliminated with the BH3 (BCL-2 homology 3 domain) mimetic ABT-263 (navitoclax) [101]. In *TP53*-mutant gastric cancer cells, a strategy utilizing the KDM4C (lysine-specific demethylase 4C) inhibitor QC6352 to induce senescence (Step 1) followed by elimination with the senolytic SSK1 (Step 2) showed significant efficacy [26].

Furthermore, this approach can be effective regardless of TP53 status. In head and neck squamous cell carcinoma, inducing senescence with cisplatin and eliminating senescent cells with navitoclax delayed tumor recurrence in both TP53-WT and TP53-mutant models [102]. Moreover, in TP53-mutant diffuse large B-cell lymphoma, the combination of doxorubicin and chidamide accelerated senescent cell apoptosis [103]. In triple-negative breast cancer, the combination of doxorubicin and the BCL-2 inhibitor venetoclax also effectively reduced the senescent cell burden regardless of TP53 status [104].

Research into next-generation senolytics is also actively expanding. Promising candidates currently under investigation include the combination of dasatinib and quercetin (D+Q), selective (BCL-xL (B-cell lymphoma extra large) inhibitors (e.g., AZD0466), peptides designed to inhibit the FOXO4 (forkhead box O4)–p53 interaction, and PP2A (protein phosphatase 2A) activators [105,106,107,108].

Despite the efficacy of first-generation senolytics such as navitoclax, their clinical translation has been hindered by on-target toxicities, including severe thrombocytopenia caused by BCL-xL inhibition in platelets. To overcome this limitation, recent research has shifted toward PROTAC (Proteolysis Targeting Chimera) technology [109]. For instance, PZ15227, a BCL-xL-targeting PROTAC, degrades BCL-xL rather than merely inhibiting it, exhibiting reduced platelet toxicity while maintaining potent senolytic activity [110].

The field of senotherapeutics from 2022 to 2025 has evolved beyond simple molecular inhibition toward a new paradigm of targeted protein degradation and pathway restoration. In particular, novel USP7 inhibitors have demonstrated multifaceted efficacy by not only inducing p53-dependent senescence and apoptosis in MDM2-overexpressing malignancies but also by remodeling the tumor microenvironment to activate immune responses and promoting bone regeneration in senile osteoporosis models [111,112,113].

Additionally, the dual degrader 753b, which leverages E3 ligases like VHL with low platelet expression, has shown breakthrough results in inhibiting NASH-to-HCC progression due to its liver-specific tropism [105,109]. Specifically, the dual degrader 753b has shown breakthrough results in inhibiting the progression of NASH-to-HCC (hepatocellular carcinoma) due to its liver-specific tropism, further solidifying the clinical feasibility of next-generation senolytics [114].

Furthermore, galacto-conjugation strategies—linking cytotoxic drugs to galactose—exploit the high lysosomal β-galactosidase activity characteristic of senescent cells [115]. This prodrug approach ensures that the toxic payload is released exclusively within senescent cells, significantly widening the therapeutic window.

To mitigate this on-target toxicity, clinical protocols are increasingly adopting an intermittent dosing (hit-and-run) schedule. This pharmacokinetic strategy exploits the slow accumulation rate of senescent cells, allowing for effective clearance with short drug exposure while permitting platelet recovery between cycles. Furthermore, the lack of p53-stratified patient selection and the absence of robust biomarkers for monitoring senescent cell burden in vivo remain major hurdles to their clinical success [100,115].

While senolytics have demonstrated robust efficacy in murine models, their translation to clinical practice faces significant hurdles due to species-specific differences in SASP composition and immune microenvironments. Consequently, validation in ongoing human trials is imperative to determine whether the impressive clearance of senescent cells observed in mice can be replicated in patients without unacceptable toxicities [40].

Moreover, a critical analysis of past failures reveals that the lack of robust pharmacodynamic biomarkers to verify senolysis in vivo has hindered clinical decision-making. Future trials must therefore prioritize the co-development of sensitive diagnostic tools, such as SASP-based liquid biopsies, to quantify therapeutic response [62,64].

### 4.3. Strategy 3: Modulating the Secretome—Senomorphics

This strategy may be particularly critical in cancers with p53 loss-of-function that secrete a pro-inflammatory SASP enriched in IL-6, IL-8, and proteases. Rapamycin (an mTOR [mechanistic target of rapamycin] inhibitor) and ruxolitinib (a JAK [Janus kinase] inhibitor) block core SASP pathways independent of p53 status [116,117]. Rapamycin selectively inhibits the translation of membrane-bound interleukin-1α to diminish NF-κB activity, effectively blunting the proinflammatory SASP [116]. By blocking this senescence-associated inflammation, rapamycin prevents senescent cells from stimulating tumor growth, underscoring its potential for treating age-related pathologies and cancer. The JAK pathway drives the SASP in senescent cells, and its inhibition effectively reduces systemic and adipose tissue inflammation [117]. Consequently, JAK inhibitors improve physical function in aged mice, highlighting their potential to alleviate age-related frailty and dysfunction.

The NF-κB and cGAS–STING pathways, key regulators of the SASP, also represent promising senomorphic targets [118]. NF-κB accumulates on senescent chromatin as a master regulator of the SASP, essential for triggering immune surveillance by NK cells. Inhibiting NF-κB allows cells to bypass senescence and develop drug resistance, demonstrating its vital tumor-suppressive role in cancer therapy outcomes [118]. Notably, the cGAS–STING pathway has been shown to amplify the SASP (particularly Type I interferons) during TIS triggered by combined PARP (poly(ADP-ribose) polymerase) and CDK4 (cyclin-dependent kinase 4)/6 inhibition [119].

### 4.4. Strategy 4: Harnessing the Immune System—Immunotherapeutic Approaches

The interplay between senescent cells and the immune system offers a pivotal therapeutic window, particularly when leveraging the concept of immunogenic senescence. TIS mediated by wild-type p53 facilitates immune clearance of tumor cells. Mechanistically, p53 responds to DNA damage by activating mitotic SENP3 (SUMO-specific protease 3), which subsequently engages cGAS signaling to stimulate potent antitumor immunity [120]. This process results in upregulation of NK cell-activating ligands, such as NKG2D-L, on the surface of senescent cells, thereby promoting their elimination [27]. Conversely, tumors with p53 deficiency fail to elicit this immunogenic response, instead establishing an immunosuppressive microenvironment that hinders immune surveillance.

To overcome the limitations imposed by p53 loss, the combination of TIS-inducing chemotherapy and ICIs, such as anti-PD-1/PD-L1 (anti–programmed cell death protein PD-1/programmed death-ligand 1) agents, has emerged as a synergistic strategy. In mouse models, this regimen significantly enhanced intratumoral T-cell infiltration and maximized antitumor efficacy, effectively counteracting the immunosuppressive nature of p53-deficient tumors [121].

Furthermore, recent advances highlight the efficacy of combining senolytics with immunotherapy to reshape the TME. Pharmacologic elimination of TIS cells using senolytics depletes immunosuppressive cell populations, including myeloid-derived suppressor cells and Tregs. Crucially, this clearance not only removes suppressive elements but also reinvigorates exhausted cytotoxic T cells, thereby restoring robust antitumor immunity [59].

In the realm of adoptive cell therapy, a novel approach targeting the urokinase plasminogen activator receptor (uPAR)—a protein specifically upregulated on senescent cells—has demonstrated breakthrough potential. CAR-T (chimeric antigen receptor T cell) cells engineered to target uPAR (uPAR–CAR-T) effectively ablated both senescent tumor cells and senescent stromal fibroblasts in preclinical models of liver and lung cancer [122]. These findings suggest that targeting senescence-associated antigens represents a viable and promising avenue for next-generation immune cell therapies. Nevertheless, the translation of senolytic CAR-T therapies to solid tumors faces hurdles, including the risks of cytokine release syndrome (CRS) and the immunosuppressive physical barriers of the TME that limit T-cell infiltration.

### 4.5. Strategy 5: Targeting p53 Mutant Weaknesses—Synthetic Lethality

The loss of functional p53 forces tumor cells to rewire their survival networks, creating reliance on alternative pathways to maintain viability. This contingency exposes specific vulnerabilities known as synthetic lethality, wherein inhibition of a compensatory pathway—harmless to normal cells—becomes lethal to p53-deficient cancer cells.

Epigenetic regulators represent a promising class of such targets. For instance, *TP53*-mutated gastric cancers exhibit critical dependency on the histone demethylase KDM4C. Targeting this dependency with the specific inhibitor QC6352 has been shown to trigger senescence, effectively serving as the first step of a one-two punch strategy that primes the tumor for subsequent elimination [26]. Similarly, in p53-mutated lung cancer, tumor survival relies on PRIM2 (DNA primase subunit 2); its inhibition also precipitates a stable senescence phenotype, further validating the induction of senescence as a viable synthetic lethal approach [123,124].

Beyond epigenetic and replication stress, defects in the DDR provide additional therapeutic windows. Since p53-mutant cancers often harbor partial defects in ATR signaling, they display heightened sensitivity to inhibition of other DNA repair factors, such as the nuclease/helicase DNA2 (DNA replication helicase/nuclease 2). Consequently, DNA2 inhibitors (e.g., d16) exert a potent synthetic lethal effect in these contexts [125]. Furthermore, metabolic adaptations offer distinct targets; p53-mutated glioblastomas, for example, develop an addiction to the Nrf2 (nuclear factor erythroid 2-related factor 2) antioxidant pathway. In these tumors, Nrf2 inhibition disrupts redox homeostasis and significantly enhances sensitivity to chemotherapy, underscoring the diverse range of synthetic lethal targets available for treating p53-deficient malignancies [126].

## 5. Conclusions and Future Perspectives

It has become increasingly evident that cellular senescence is not merely a terminal endpoint of cancer therapy but rather a plastic state that modulates therapeutic responsiveness via secretome evolution. Strategies that reactivate the p53 pathway to induce senescence, eliminate established therapy-induced senescent (TIS) cells via senolytics, or modulate their proteotoxic effects through senomorphics represent innovative avenues to maximize the efficacy of conventional anticancer regimens while minimizing adverse effects. However, successful translation of these strategies into clinical practice necessitates addressing several key challenges.

**Figure 6:**
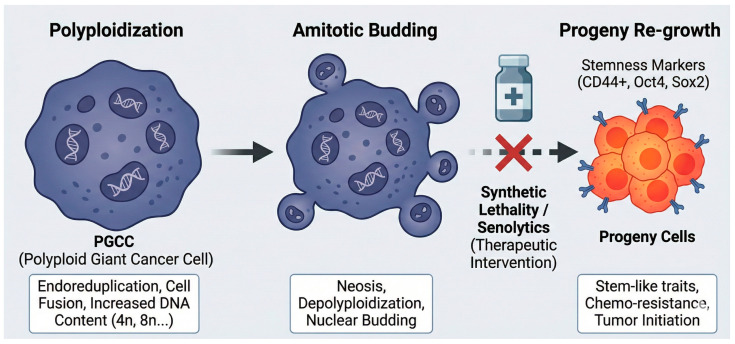
Molecular Mechanisms of Senescence Escape and Progeny Re-growth via Polyploid Giant Cancer Cells (PGCCs). Polyploidization: Under therapeutic stress or environmental cues, cancer cells undergo endoreduplication or cell fusion, leading to the formation of Polyploid Giant Cancer Cells (PGCCs). These cells are characterized by significantly increased DNA content (4n, 8n, …). Amitotic Budding: Rather than conventional mitosis, PGCCs escape senescence through a specialized cell division process known as neosis. This involves depolyploidization and nuclear budding, where the giant nucleus generates multiple small bud-like structures. Progeny Re-growth: The resulting progeny cells re-acquire stem-like traits and express key stemness markers such as CD44^+^, Oct4, and Sox2. These cells are highly aggressive, exhibiting enhanced chemo-resistance and tumor initiation capabilities, which drive cancer recurrence. Therapeutic Intervention: Therapeutic Intervention: The red ‘X’ symbol indicates the blockade of the transition from PGCCs to progeny re-growth. The transition from PGCC-mediated senescence to progeny re-growth represents a critical window for treatment. Targeted interventions using synthetic lethality or senolytic agents aim to block this escape mechanism, potentially preventing tumor relapse and overcoming drug resistance.

Foremost among these is the implementation of personalized therapy tailored to p53 status. The functional state of p53 within a patient’s tumor—whether wild-type, deficient, harboring GOF mutations, or exhibiting MDM2 overexpression—serves as a pivotal biomarker guiding the selection of senescence-targeting modalities. For instance, tumors with MDM2 overexpression may be optimally targeted by p53 activators such as Nutlins. Conversely, p53-wild-type tumors may benefit most from TIS induction followed by immunotherapy, whereas p53-deficient or mutant tumors may require a strategy involving TIS induction followed immediately by senolytics or senomorphics, or exploitation of synthetic lethal vulnerabilities such as DNA2 or KDM4C inhibition (Figure 6).

Additionally, achieving therapeutic specificity remains a critical hurdle; agents must distinguish between senescent tumor cells and physiological senescence in normal tissues to avoid off-target toxicity. Furthermore, reliable biomarkers capable of quantifying the burden of TIS cells and SASP activity in vivo are urgently needed.

Recent advances in clinical senescence biomarkers (2022–2025) have focused on circulating SASP proteins (e.g., GDF15, IL-6, TNFR1, VEGFA) as non-invasive markers of senescent cell burden, predicting mortality, mobility disability, and other age-related outcomes [127,128]. Panels including GDF15 and IL-6 improved outcome prediction in the Health ABC cohort, while GDF15, RAGE, and VEGFA were linked to mortality and proposed as biological age indicators [127,128]. T-cell p16^INK4a^ variant 5 expression assesses senescence burden and predicts senolytic response to dasatinib plus quercetin [129]. Emerging studies validate GDF15 and extracellular vesicle signatures as senescent burden proxies, with early clinical promise [130]. Senolytic trials employ T-cell p16 and SASP panels for efficacy monitoring, and the LIFE study showed biomarker links to mobility disability reducible by physical activity [129,131]. Integrating liquid biopsy markers is essential for senolytic pharmacodynamic evaluation. Despite promise, heterogeneity and specificity challenges require further large-scale validation [127]. These advances support the geroscience hypothesis and age-related disease prevention.

Finally, comprehensive treatment protocols must be established to determine optimal timing, patient selection criteria, and combinatorial regimens with standard-of-care therapies. Addressing these challenges will position the modulation of cellular senescence as a key strategy for unlocking a new era of personalized cancer therapy, effectively breaking the cycle of recurrence and resistance while minimizing therapeutic side effects.

To bridge the remaining clinical gaps and transition from bench to bedside, the focus must shift from general senescence induction to the precise eradication of detrimental cell populations. Looking forward, the next decade of senescence-targeted therapy must overcome three primary barriers: (1) establishing quantitative in vivo biomarkers for monitoring TIS, (2) developing tissue-specific senomodulators (e.g., BCL-xL PROTACs) to minimize systemic toxicities, and (3) implementing p53-stratified clinical protocols. Our proposed framework emphasizes a one-two punch strategy where TIS induction is followed by personalized senolysis to ensure complete tumor clearance and prevent aggressive escape phenotypes.

## Data Availability

No new data were created or analyzed in this study. Data sharing is not applicable to this article.

## Abbreviations

AMPK	AMP-activated Protein Kinase 1
ATM	Ataxia Telangiectasia Mutated 2
ATR	Ataxia Telangiectasia and Rad3-related
CAR-T	Chimeric Antigen Receptor T-cell
CTL	Cytotoxic T Lymphocyte
DDR	DNA Damage Response
DLBCL	Diffuse Large B-cell Lymphoma
DSB	DNA Double-Strand Break
EMT	Epithelial–Mesenchymal Transition
GDF15	Growth Differentiation Factor 15
GOF	Gain-of-Function
HCC	Hepatocellular Carcinoma
HNSCC	Head and Neck Squamous Cell Carcinoma
ICD	Immunogenic Cell Death
ICI	Immune Checkpoint Inhibitor
MDSC	Myeloid-Derived Suppressor Cell
MMP	Matrix Metalloproteinase
NED	Neuroendocrine Differentiation
NK	Natural Killer
OCCC	Ovarian Clear Cell Carcinoma
OIS	Oncogene-Induced Senescence
PNI	Perineural Invasion
PROTAC	Proteolysis Targeting Chimera
ROS	Reactive Oxygen Species
SA-β-gal	Senescence-Associated β-galactosidase
SAHF	Senescence-Associated Heterochromatin Foci
SASP	Senescence-Associated Secretory Phenotype
TASc	Tumor-Associated Schwann Cell
TIS	Therapy-Induced Senescence
TME	Tumor Microenvironment
TNBC	Triple-Negative Breast Cancer
Treg	Regulatory T cell
uPAR	Urokinase Plasminogen Activator Receptor
VNS	Vagus Nerve Stimulation

## References

[1] Khan S.U., Fatima K., Aisha S., Malik F. (2024). Unveiling the mechanisms and challenges of cancer drug resistance. Cell Commun. Signal..

[2] Vasan N., Baselga J., Hyman D.M. (2019). A view on drug resistance in cancer. Nature.

[3] López-Otín C., Blasco M.A., Partridge L., Serrano M., Kroemer G. (2023). Hallmarks of aging: An expanding universe. Cell.

[4] Fitsiou E., Soto-Gamez A., Demaria M. (2022). Biological functions of therapy-induced senescence in cancer. Seminars in Cancer Biology.

[5] Winkler F., Venkatesh H.S., Amit M., Batchelor T., Demir I.E., Deneen B., Gutmann D.H., Hervey-Jumper S., Kuner T., Mabbott D. (2023). Cancer neuroscience: State of the field, emerging directions. Cell.

[6] Al Mamun A., Sufian M.A., Uddin M.S., Sumsuzzman D.M., Jeandet P., Islam M.S., Zhang H.-J., Kong A.-N., Sarwar M.S. (2022). Exploring the role of senescence inducers and senotherapeutics as targets for anticancer natural products. Eur. J. Pharmacol..

[7] D’adda Di Fagagna F. (2008). Living on a break: Cellular senescence as a DNA-damage response. Nat. Rev. Cancer.

[8] Levine A.J. (2020). p53: 800 million years of evolution and 40 years of discovery. Nat. Rev. Cancer.

[9] Wang H., Guo M., Wei H., Chen Y. (2023). Targeting p53 pathways: Mechanisms, structures and advances in therapy. Signal Transduct. Target. Ther..

[10] Serrano M., Blasco M.A. (2007). Cancer and ageing: Convergent and divergent mechanisms. Nat. Rev. Mol. Cell Biol..

[11] Piano A., Titorenko V.I. (2014). The intricate interplay between mechanisms underlying aging and cancer. Aging Dis..

[12] Liu B., Peng Z., Zhang H., Zhang N., Liu Z., Xia Z., Huang S., Luo P., Cheng Q. (2025). Regulation of cellular senescence in tumor progression and therapeutic targeting: Mechanisms and pathways. Mol. Cancer.

[13] Haupt Y., Maya R., Kazaz A., Oren M. (1997). Mdm2 promotes the rapid degradation of p53. Nature.

[14] Kubbutat M.H., Jones S.N., Vousden K.H. (1997). Regulation of p53 stability by Mdm2. Nature.

[15] Wang W., Albadari N., Du Y., Fowler J.F., Sang H.T., Xian W., McKeon F., Li W., Zhou J., Zhang R. (2024). MDM2 inhibitors for cancer therapy: The past, present, and future. Pharmacol. Rev..

[16] Li M., Brooks C.L., Wu-Baer F., Chen D., Baer R., Gu W. (2003). Mono-versus polyubiquitination: Differential control of p53 fate by Mdm2. Science.

[17] Grier J.D., Xiong S., Elizondo-Fraire A.C., Parant J.M., Lozano G. (2006). Tissue-specific differences of p53 inhibition by Mdm2 and Mdm4. Mol. Cell. Biol..

[18] Xiong S., Zhang Y., Zhou X., Pant V., Mirani A., Gencel-Augusto J., Chau G., You M.J., Lozano G. (2025). Dependence on Mdm2 for Mdm4 inhibition of p53 activity. Cancer Lett..

[19] Luo J., Nikolaev A.Y., Imai S.-I., Chen D., Su F., Shiloh A., Guarente L., Gu W. (2001). Negative control of p53 by Sir2α promotes cell survival under stress. Cell.

[20] Ong A.L., Ramasamy T.S. (2018). Role of Sirtuin1-p53 regulatory axis in aging, cancer and cellular reprogramming. Ageing Res. Rev..

[21] Pomerantz J., Schreiber-Agus N., Liégeois N.J., Silverman A., Alland L., Chin L., Potes J., Chen K., Orlow I., Lee H.-W. (1998). The Ink4a tumor suppressor gene product, p19Arf, interacts with MDM2 and neutralizes MDM2’s inhibition of p53. Cell.

[22] Sherr C.J. (2001). The INK4a/ARF network in tumour suppression. Nat. Rev. Mol. Cell Biol..

[23] Ajoolabady A., Pratico D., Bahijri S., Tuomilehto J., Uversky V.N., Ren J. (2025). Hallmarks of cellular senescence: Biology, mechanisms, regulations. Exp. Mol. Med..

[24] Kuo M.-L., Duncavage E.J., Mathew R., Den Besten W., Pei D., Naeve D., Yamamoto T., Cheng C., Sherr C.J., Roussel M.F. (2003). Arf induces p53-dependent and-independent antiproliferative genes. Cancer Res..

[25] Gu W., Roeder R.G. (1997). Activation of p53 sequence-specific DNA binding by acetylation of the p53 C-terminal domain. Cell.

[26] Wang K., Gong Z., Chen Y., Zhang M., Wang S., Yao S., Liu Z., Huang Z., Fei B. (2023). KDM4C-mediated senescence defense is a targetable vulnerability in gastric cancer harboring TP53 mutations. Clin. Epigenetics.

[27] Soriani A., Zingoni A., Cerboni C., Iannitto M.L., Ricciardi M.R., Di Gialleonardo V., Cippitelli M., Fionda C., Petrucci M.T., Guarini A. (2009). ATM-ATR–dependent up-regulation of DNAM-1 and NKG2D ligands on multiple myeloma cells by therapeutic agents results in enhanced NK-cell susceptibility and is associated with a senescent phenotype. Blood J. Am. Soc. Hematol..

[28] Serrano M., Lin A.W., McCurrach M.E., Beach D., Lowe S.W. (1997). Oncogenic ras provokes premature cell senescence associated with accumulation of p53 and p16INK4a. Cell.

[29] Kuilman T., Michaloglou C., Mooi W.J., Peeper D.S. (2010). The essence of senescence. Genes Dev..

[30] Guo J., Huang X., Dou L., Yan M., Shen T., Tang W., Li J. (2022). Aging and aging-related diseases: From molecular mechanisms to interventions and treatments. Signal Transduct. Target. Ther..

[31] Rodier F., Campisi J. (2011). Four faces of cellular senescence. J. Cell Biol..

[32] Gorgoulis V., Adams P.D., Alimonti A., Bennett D.C., Bischof O., Bishop C., Campisi J., Collado M., Evangelou K., Ferbeyre G. (2019). Cellular senescence: Defining a path forward. Cell.

[33] Dimri G.P., Lee X., Basile G., Acosta M., Scott G., Roskelley C., Medrano E.E., Linskens M., Rubelj I., Pereira-Smith O. (1995). A biomarker that identifies senescent human cells in culture and in aging skin in vivo. Proc. Natl. Acad. Sci. USA.

[34] Suryadevara V., Hudgins A.D., Rajesh A., Pappalardo A., Karpova A., Dey A.K., Hertzel A., Agudelo A., Rocha A., Soygur B. (2024). SenNet recommendations for detecting senescent cells in different tissues. Nat. Rev. Mol. Cell Biol..

[35] Narita M., Nuñez S., Heard E., Narita M., Lin A.W., Hearn S.A., Spector D.L., Hannon G.J., Lowe S.W. (2003). Rb-mediated heterochromatin formation and silencing of E2F target genes during cellular senescence. Cell.

[36] Wang B., Han J., Elisseeff J.H., Demaria M. (2024). The senescence-associated secretory phenotype and its physiological and pathological implications. Nat. Rev. Mol. Cell Biol..

[37] Krizhanovsky V., Yon M., Dickins R.A., Hearn S., Simon J., Miething C., Yee H., Zender L., Lowe S.W. (2008). Senescence of activated stellate cells limits liver fibrosis. Cell.

[38] Marin I., Boix O., Garcia-Garijo A., Sirois I., Caballe A., Zarzuela E., Ruano I., Attolini C.S.-O., Prats N., López-Domínguez J.A. (2023). Cellular senescence is immunogenic and promotes antitumor immunity. Cancer Discov..

[39] Baker D.J., Wijshake T., Tchkonia T., LeBrasseur N.K., Childs B.G., Van De Sluis B., Kirkland J.L., Van Deursen J.M. (2011). Clearance of p16Ink4a-positive senescent cells delays ageing-associated disorders. Nature.

[40] Chaib S., Tchkonia T., Kirkland J.L. (2022). Cellular senescence and senolytics: The path to the clinic. Nat. Med..

[41] Harley C.B., Futcher A.B., Greider C.W. (1990). Telomeres shorten during ageing of human fibroblasts. Nature.

[42] Hoare M., Ito Y., Kang T.-W., Weekes M.P., Matheson N.J., Patten D.A., Shetty S., Parry A.J., Menon S., Salama R. (2016). NOTCH1 mediates a switch between two distinct secretomes during senescence. Nat. Cell Biol..

[43] Pribluda A., Elyada E., Wiener Z., Hamza H., Goldstein R.E., Biton M., Burstain I., Morgenstern Y., Brachya G., Billauer H. (2013). A senescence-inflammatory switch from cancer-inhibitory to cancer-promoting mechanism. Cancer Cell.

[44] Cohn R.L., Gasek N.S., Kuchel G.A., Xu M. (2023). The heterogeneity of cellular senescence: Insights at the single-cell level. Trends Cell Biol..

[45] Theodorakis N., Feretzakis G., Tzelves L., Paxinou E., Hitas C., Vamvakou G., Verykios V.S., Nikolaou M. (2024). Integrating machine learning with multi-omics technologies in geroscience: Towards personalized medicine. J. Pers. Med..

[46] Sanborn M.A., Wang X., Gao S., Dai Y., Rehman J. (2025). Unveiling the cell-type-specific landscape of cellular senescence through single-cell transcriptomics using SenePy. Nat. Commun..

[47] Coppé J.-P., Patil C.K., Rodier F., Sun Y., Muñoz D.P., Goldstein J., Nelson P.S., Desprez P.-Y., Campisi J. (2008). Senescence-associated secretory phenotypes reveal cell-nonautonomous functions of oncogenic RAS and the p53 tumor suppressor. PLoS Biol..

[48] Loo T.M., Miyata K., Tanaka Y., Takahashi A. (2020). Cellular senescence and senescence-associated secretory phenotype via the cGAS-STING signaling pathway in cancer. Cancer Sci..

[49] Li T., Chen Z.J. (2018). The cGAS–cGAMP–STING pathway connects DNA damage to inflammation, senescence, and cancer. J. Exp. Med..

[50] Paul P., Kumar A., Parida A.S., De A.K., Bhadke G., Khatua S., Tiwari B. (2025). p53-mediated regulation of LINE1 retrotransposon-derived R-loops. J. Biol. Chem..

[51] De Cecco M., Ito T., Petrashen A.P., Elias A.E., Skvir N.J., Criscione S.W., Caligiana A., Brocculi G., Adney E.M., Boeke J.D. (2019). L1 drives IFN in senescent cells and promotes age-associated inflammation. Nature.

[52] Mathavarajah S., Dellaire G. (2023). LINE-1: An emerging initiator of cGAS-STING signalling and inflammation that is dysregulated in disease. Biochem. Cell Biol..

[53] Lujambio A., Akkari L., Simon J., Grace D., Tschaharganeh D.F., Bolden J.E., Zhao Z., Thapar V., Joyce J.A., Krizhanovsky V. (2013). Non-cell-autonomous tumor suppression by p53. Cell.

[54] Chen Z., Huang L., Ding L., Zhang C., Li Y., Wang B., Shi J., Zhang J. (2025). NFATc1 facilitates hepatocellular carcinoma progression by regulating the senescence-associated secretory phenotype. Sci. Rep..

[55] Gilbert L.A., Hemann M.T. (2010). DNA damage-mediated induction of a chemoresistant niche. Cell.

[56] Ling Y.-W., Duan J.-L., Jiang Z.-J., Yang Z., Liu J.-J., Song P., Fang Z.-Q., Yue Z.-S., He F., Dou K.-F. (2025). Diabetes reshapes pancreatic cancer-associated endothelial niche by accelerating senescence. Nat. Commun..

[57] Krtolica A., Parrinello S., Lockett S., Desprez P.-Y., Campisi J. (2001). Senescent fibroblasts promote epithelial cell growth and tumorigenesis: A link between cancer and aging. Proc. Natl. Acad. Sci. USA.

[58] Sahai E., Astsaturov I., Cukierman E., DeNardo D.G., Egeblad M., Evans R.M., Fearon D., Greten F.R., Hingorani S.R., Hunter T. (2020). A framework for advancing our understanding of cancer-associated fibroblasts. Nat. Rev. Cancer.

[59] Ruhland M.K., Loza A.J., Capietto A.-H., Luo X., Knolhoff B.L., Flanagan K.C., Belt B.A., Alspach E., Leahy K., Luo J. (2016). Stromal senescence establishes an immunosuppressive microenvironment that drives tumorigenesis. Nat. Commun..

[60] Wiklund F.E., Bennet A.M., Magnusson P.K., Eriksson U.K., Lindmark F., Wu L., Yaghoutyfam N., Marquis C.P., Stattin P., Pedersen N.L. (2010). Macrophage inhibitory cytokine-1 (MIC-1/GDF15): A new marker of all-cause mortality. Aging Cell.

[61] Pence B.D., Yarbro J.R., Emmons R.S. (2021). Growth differentiation factor-15 is associated with age-related monocyte dysfunction. Aging Med..

[62] Arif M., Lehoczki A., Haskó G., Lohoff F.W., Ungvari Z., Pacher P. (2025). Global and tissue-specific transcriptomic dysregulation in human aging: Pathways and predictive biomarkers. GeroScience.

[63] Roth P., Junker M., Tritschler I., Mittelbronn M., Dombrowski Y., Breit S.N., Tabatabai G., Wick W., Weller M., Wischhusen J. (2010). GDF-15 contributes to proliferation and immune escape of malignant gliomas. Clin. Cancer Res..

[64] Lyu Z., Fan N., Deng P., Huang Q., Bruns C., Zhao Y. (2025). GDF15 connecting ageing and cancer: Mechanistic insights and therapeutic opportunities. Ageing Cancer Res. Treat..

[65] Brem S. (2024). Vagus nerve stimulation: Novel concept for the treatment of glioblastoma and solid cancers by cytokine (interleukin-6) reduction, attenuating the SASP, enhancing tumor immunity. Brain Behav. Immun.-Health.

[66] Eom Y., Lee S.H. (2025). Xylene Impairs Neuronal Development by Dysregulating Calcium Homeostasis and Neuronal Activity in Developing Hippocampal Neurons. Biomol. Ther..

[67] Carreno G., Guiho R., Martinez-Barbera J.P. (2021). Cell senescence in neuropathology: A focus on neurodegeneration and tumours. Neuropathol. Appl. Neurobiol..

[68] Huang L., Qi G., Chen G., Duan J., Dai C., Lu Y., Zhou Q. (2025). Tumor-associated Schwann cells as new therapeutic target in non-neurological cancers. Cancer Lett..

[69] Yin Y., Zhou Y., Zhou J., Zhao L., Hu H., Xiao M., Niu B., Peng J., Dai Y., Tang Y. (2023). Cisplatin causes erectile dysfunction by decreasing endothelial and smooth muscle content and inducing cavernosal nerve senescence in rats. Front. Endocrinol..

[70] Raynard C., Ma X., Huna A., Tessier N., Massemin A., Zhu K., Flaman J.m., Moulin F., Goehrig D., Medard J.j. (2022). NF-κB-dependent secretome of senescent cells can trigger neuroendocrine transdifferentiation of breast cancer cells. Aging Cell.

[71] Nguyen T.M., Ngoc D.T.M., Choi J.-H., Lee C.-H. (2023). Unveiling the neural environment in cancer: Exploring the role of neural circuit players and potential therapeutic strategies. Cells.

[72] Lee C., Cho J., Lee K. (2020). Tumour regression via integrative regulation of neurological, inflammatory, and hypoxic tumour microenvironment. Biomol. Ther..

[73] Bhat S., Adiga D., Shukla V., Guruprasad K.P., Kabekkodu S.P., Satyamoorthy K. (2022). Metastatic suppression by DOC2B is mediated by inhibition of epithelial-mesenchymal transition and induction of senescence. Cell Biol. Toxicol..

[74] Chen J. (2016). The cell-cycle arrest and apoptotic functions of p53 in tumor initiation and progression. Cold Spring Harb. Perspect. Med..

[75] Cho J. (2025). Understanding tumor dormancy: From experimental models to mechanisms and therapeutic strategies. Biomol. Ther..

[76] Yang H., Zhang K., Guo Y., Guo X., Hou K., Hou J., Luo Y., Liu J., Jia S. (2023). Gain-of-Function p53N236S Mutation Drives the Bypassing of HRasV12-Induced Cellular Senescence via PGC–1α. Int. J. Mol. Sci..

[77] Collado M., Serrano M. (2010). Senescence in tumours: Evidence from mice and humans. Nat. Rev. Cancer.

[78] Fischer M., Quaas M., Steiner L., Engeland K. (2016). The p53-p21-DREAM-CDE/CHR pathway regulates G2/M cell cycle genes. Nucleic Acids Res..

[79] Joruiz S.M., Von Muhlinen N., Horikawa I., Gilbert M.R., Harris C.C. (2024). Distinct functions of wild-type and R273H mutant Δ133p53α differentially regulate glioblastoma aggressiveness and therapy-induced senescence. Cell Death Dis..

[80] Chen C., Chen J., Zhang Y., Zhang Q., Shi H. (2025). Senescence-associated secretory phenotype in lung cancer: Remodeling the tumor microenvironment for metastasis and immune suppression. Front. Oncol..

[81] Rehman S.K., Haynes J., Collignon E., Brown K.R., Wang Y., Nixon A.M., Bruce J.P., Wintersinger J.A., Mer A.S., Lo E.B. (2021). Colorectal cancer cells enter a diapause-like DTP state to survive chemotherapy. Cell.

[82] Zhou X., Zhou M., Zheng M., Tian S., Yang X., Ning Y., Li Y., Zhang S. (2022). Polyploid giant cancer cells and cancer progression. Front. Cell Dev. Biol..

[83] Sikora E., Czarnecka-Herok J., Bojko A., Sunderland P. (2022). Therapy-induced polyploidization and senescence: Coincidence or interconnection?. Seminars in Cancer Biology.

[84] Saleh T., Tyutyunyk-Massey L., Murray G.F., Alotaibi M.R., Kawale A.S., Elsayed Z., Henderson S.C., Yakovlev V., Elmore L.W., Toor A. (2019). Tumor cell escape from therapy-induced senescence. Biochem. Pharmacol..

[85] Yang J.-H., Petty C.A., Dixon-McDougall T., Lopez M.V., Tyshkovskiy A., Maybury-Lewis S., Tian X., Ibrahim N., Chen Z., Griffin P.T. (2023). Chemically induced reprogramming to reverse cellular aging. Aging.

[86] Browder K.C., Reddy P., Yamamoto M., Haghani A., Guillen I.G., Sahu S., Wang C., Luque Y., Prieto J., Shi L. (2022). In vivo partial reprogramming alters age-associated molecular changes during physiological aging in mice. Nat. Aging.

[87] Haraoka Y., Akieda Y., Nagai Y., Mogi C., Ishitani T. (2022). Zebrafish imaging reveals TP53 mutation switching oncogene-induced senescence from suppressor to driver in primary tumorigenesis. Nat. Commun..

[88] Mahat D.B., Kumra H., Castro S.A., Metcalf E., Nguyen K., Morisue R., Ho W.W., Chen I., Sullivan B., Yim L.H. (2025). Mutant p53 exploits enhancers to elevate immunosuppressive chemokine expression and impair immune checkpoint inhibitors in pancreatic cancer. Immunity.

[89] Liu K., Garan L.A.W., Lin F.-T., Lin W.-C. (2025). Mutant p53 variants differentially impact replication initiation and activate cGAS-STING to affect immune checkpoint inhibition. Commun. Biol..

[90] Wang Z., Shi M., Liu B., Zhang X., Lin W., Yang Y., Huang Z., Yang D., Chu T., Zheng D. (2025). Low-dose statins restore innate immune response in breast cancer cells via suppression of mutant p53. Front. Pharmacol..

[91] Dixon S.J., Stockwell B.R. (2014). The role of iron and reactive oxygen species in cell death. Nat. Chem. Biol..

[92] Su L.-J., Zhang J.-H., Gomez H., Murugan R., Hong X., Xu D., Jiang F., Peng Z.-Y. (2019). Reactive oxygen species-induced lipid peroxidation in apoptosis, autophagy, and ferroptosis. Oxidative Med. Cell. Longev..

[93] Jung K.H., Kim S.E., Go H.G., Lee Y.J., Park M.S., Ko S., Han B.S., Yoon Y.-C., Cho Y.J., Lee P. (2023). Synergistic renoprotective effect of melatonin and zileuton by inhibition of ferroptosis via the AKT/mTOR/NRF2 signaling in kidney injury and fibrosis. Biomol. Ther..

[94] Liu Y., Gu W. (2022). p53 in ferroptosis regulation: The new weapon for the old guardian. Cell Death Differ..

[95] Toledo F., Wahl G.M. (2007). MDM2 and MDM4: P53 regulators as targets in anticancer therapy. Int. J. Biochem. Cell Biol..

[96] Vassilev L.T., Vu B.T., Graves B., Carvajal D., Podlaski F., Filipovic Z., Kong N., Kammlott U., Lukacs C., Klein C. (2004). In vivo activation of the p53 pathway by small-molecule antagonists of MDM2. Science.

[97] Shangary S., Wang S. (2009). Small-molecule inhibitors of the MDM2-p53 protein-protein interaction to reactivate p53 function: A novel approach for cancer therapy. Annu. Rev. Pharmacol. Toxicol..

[98] Saleh M.N., Patel M.R., Bauer T.M., Goel S., Falchook G.S., Shapiro G.I., Chung K.Y., Infante J.R., Conry R.M., Rabinowits G. (2021). Phase 1 trial of ALRN-6924, a dual inhibitor of MDMX and MDM2, in patients with solid tumors and lymphomas bearing wild-type TP53. Clin. Cancer Res..

[99] Michaeli O., Luz I., Vatarescu M., Manko T., Weizman N., Korotinsky Y., Tsitrina A., Braiman A., Arazi L., Cooks T. (2024). APR-246 as a radiosensitization strategy for mutant p53 cancers treated with alpha-particles-based radiotherapy. Cell Death Dis..

[100] Yosef R., Pilpel N., Tokarsky-Amiel R., Biran A., Ovadya Y., Cohen S., Vadai E., Dassa L., Shahar E., Condiotti R. (2016). Directed elimination of senescent cells by inhibition of BCL-W and BCL-XL. Nat. Commun..

[101] Ito Y., Nakamura K., Nakagawa-Saito Y., Takenouchi S., Suzuki S., Togashi K., Sugai A., Mitobe Y., Seino M., Ohta T. (2025). Senolytic Elimination of Senescent Ovarian Clear Cell Carcinoma Cells Induced by CEP-1347 with the BH3 Mimetic Navitoclax. Anticancer Res..

[102] Osman A.A., Monroe M.M., Ortega Alves M.V., Patel A.A., Katsonis P., Fitzgerald A.L., Neskey D.M., Frederick M.J., Woo S.H., Caulin C. (2015). Wee-1 kinase inhibition overcomes cisplatin resistance associated with high-risk TP53 mutations in head and neck cancer through mitotic arrest followed by senescence. Mol. Cancer Ther..

[103] Yin Y.Y., Liu Y., Lu Y.Q., Tang Z.M., Zhu Z.X., Zhu M.Y., Chen H.Y., Hui H., Xu J.Y., Li H. (2025). Chidamide Accelerates the Death of Senescence-Like Diffuse Large B-Cell Lymphoma Cells with TP53 Mutation Induced by Doxorubicin. FASEB J..

[104] Schreiber A.R., Smoots S.G., Jackson M.M., Bagby S.M., Dus E.D., Dominguez A.T., Binns C.A., Pitts T.M., Diamond J.R. (2025). Potentiating doxorubicin activity through BCL-2 inhibition in p53 wild-type and mutated triple-negative breast cancer. Front. Oncol..

[105] Novais E.J., Tran V.A., Johnston S.N., Darris K.R., Roupas A.J., Sessions G.A., Shapiro I.M., Diekman B.O., Risbud M.V. (2021). Long-term treatment with senolytic drugs Dasatinib and Quercetin ameliorates age-dependent intervertebral disc degeneration in mice. Nat. Commun..

[106] Patterson C.M., Balachander S.B., Grant I., Pop-Damkov P., Kelly B., McCoull W., Parker J., Giannis M., Hill K.J., Gibbons F.D. (2021). Design and optimisation of dendrimer-conjugated Bcl-2/xL inhibitor, AZD0466, with improved therapeutic index for cancer therapy. Commun. Biol..

[107] Kang D., Lim Y., Ahn D., Lee J., Park C.-J. (2025). Peptide inhibitors targeting FOXO4-p53 interactions and inducing senescent cancer cell-specific apoptosis. J. Med. Chem..

[108] Peris I., Romero-Murillo S., Martinez-Balsalobre E., Farrington C.C., Arriazu E., Marcotegui N., Jimenez-Munoz M., Alburquerque-Prieto C., Torres-Lopez A., Fresquet V. (2023). Activation of the PP2A-B56alpha heterocomplex synergizes with venetoclax therapies in AML through BCL2 and MCL1 modulation. Blood.

[109] Ma Z., Zhou J. (2025). NDA submission of vepdegestrant (ARV-471) to US FDA: The beginning of a new era of PROTAC degraders. J. Med. Chem..

[110] He Y., Zhang X., Chang J., Kim H.-N., Zhang P., Wang Y., Khan S., Liu X., Zhang X., Lv D. (2020). Using proteolysis-targeting chimera technology to reduce navitoclax platelet toxicity and improve its senolytic activity. Nat. Commun..

[111] He Y., Li W., Lv D., Zhang X., Zhang X., Ortiz Y.T., Budamagunta V., Campisi J., Zheng G., Zhou D. (2020). Inhibition of USP7 activity selectively eliminates senescent cells in part via restoration of p53 activity. Aging Cell.

[112] Zhou F., Wang Z., Li H., Wang D., Wu Z., Bai F., Wang Q., Luo W., Zhang G., Xiong Y. (2025). USP7 inhibition promotes early osseointegration in senile osteoporotic mice. J. Dent. Res..

[113] Korenev G., Yakukhnov S., Druk A., Golovina A., Chasov V., Mirgayazova R., Ivanov R., Bulatov E. (2022). USP7 inhibitors in cancer immunotherapy: Current status and perspective. Cancers.

[114] Yang Y., Jn-Simon N., He Y., Sun C., Zhang P., Hu W., Tian T., Zeng H., Basha S., Huerta A.S. (2025). A BCL-xL/BCL-2 PROTAC effectively clears senescent cells in the liver and reduces MASH-driven hepatocellular carcinoma in mice. Nat. Aging.

[115] González-Gualda E., Pàez-Ribes M., Lozano-Torres B., Macias D., Wilson J.R., González-López C., Ou H.L., Mirón-Barroso S., Zhang Z., Lérida-Viso A. (2020). Galacto-conjugation of Navitoclax as an efficient strategy to increase senolytic specificity and reduce platelet toxicity. Aging Cell.

[116] Laberge R.-M., Sun Y., Orjalo A.V., Patil C.K., Freund A., Zhou L., Curran S.C., Davalos A.R., Wilson-Edell K.A., Liu S. (2015). MTOR regulates the pro-tumorigenic senescence-associated secretory phenotype by promoting IL1A translation. Nat. Cell Biol..

[117] Xu M., Tchkonia T., Ding H., Ogrodnik M., Lubbers E.R., Pirtskhalava T., White T.A., Johnson K.O., Stout M.B., Mezera V. (2015). JAK inhibition alleviates the cellular senescence-associated secretory phenotype and frailty in old age. Proc. Natl. Acad. Sci. USA.

[118] Chien Y., Scuoppo C., Wang X., Fang X., Balgley B., Bolden J.E., Premsrirut P., Luo W., Chicas A., Lee C.S. (2011). Control of the senescence-associated secretory phenotype by NF-κB promotes senescence and enhances chemosensitivity. Genes. Dev..

[119] Wang T., Liu W., Shen Q., Tao R., Li C., Shen Q., Lin Y., Huang Y., Yang L., Xie G. (2023). Combination of PARP inhibitor and CDK4/6 inhibitor modulates cGAS/STING-dependent therapy-induced senescence and provides “one-two punch” opportunity with anti-PD-L1 therapy in colorectal cancer. Cancer Sci..

[120] Hu G., Chen Y., Yang X., Wang Y., He J., Wang T., Fan Q., Deng L., Tu J., Tan H. (2022). Mitotic SENP3 activation couples with cGAS signaling in tumor cells to stimulate anti-tumor immunity. Cell Death Dis..

[121] Ruscetti M., Leibold J., Bott M.J., Fennell M., Kulick A., Salgado N.R., Chen C.-C., Ho Y.-J., Sanchez-Rivera F.J., Feucht J. (2018). NK cell–mediated cytotoxicity contributes to tumor control by a cytostatic drug combination. Science.

[122] Amor C., Feucht J., Leibold J., Ho Y.-J., Zhu C., Alonso-Curbelo D., Mansilla-Soto J., Boyer J.A., Li X., Giavridis T. (2020). Senolytic CAR T cells reverse senescence-associated pathologies. Nature.

[123] Srihari S., Singla J., Wong L., Ragan M.A. (2015). Inferring synthetic lethal interactions from mutual exclusivity of genetic events in cancer. Biol. Direct.

[124] Wang T., Tang T., Jiang Y., He T., Qi L., Chang H., Qiao Y., Sun M., Shan C., Zhu X. (2022). PRIM2 promotes cell cycle and tumor progression in p53-mutant lung cancer. Cancers.

[125] Folly-Kossi H., Graves J.D., Garan L.A.W., Lin F.-T., Lin W.-C. (2023). DNA2 nuclease inhibition confers synthetic lethality in cancers with mutant p53 and synergizes with PARP inhibitors. Cancer Res. Commun..

[126] DeNicola G.M., Karreth F.A., Humpton T.J., Gopinathan A., Wei C., Frese K., Mangal D., Yu K.H., Yeo C.J., Calhoun E.S. (2011). Oncogene-induced Nrf2 transcription promotes ROS detoxification and tumorigenesis. Nature.

[127] Cummings S.R., Lui L.-Y., Zaira A., Mau T., Fielding R.A., Atkinson E.J., Patel S., LeBrasseur N. (2025). Biomarkers of cellular senescence and major health outcomes in older adults. Geroscience.

[128] St. Sauver J.L., Weston S.A., Atkinson E.J., Mc Gree M.E., Mielke M.M., White T.A., Heeren A.A., Olson J.E., Rocca W.A., Palmer A.K. (2023). Biomarkers of cellular senescence and risk of death in humans. Aging Cell.

[129] Farr J.N., Monroe D.G., Atkinson E.J., Froemming M.N., Ruan M., LeBrasseur N.K., Khosla S. (2025). Characterization of human senescent cell biomarkers for clinical trials. Aging Cell.

[130] Fukumoto T., Shimosawa T., Yakabe M., Yoshida S., Yoshida Y. (2025). Recent advances in biomarkers for senescence: Bridging basic research to clinic. Geriatr. Gerontol. Int..

[131] Fielding R.A., Atkinson E.J., Aversa Z., White T.A., Heeren A.A., Achenbach S.J., Mielke M.M., Cummings S.R., Pahor M., Leeuwenburgh C. (2022). Associations between biomarkers of cellular senescence and physical function in humans: Observations from the lifestyle interventions for elders (LIFE) study. Geroscience.

